# A Comparative Study of Cluster Detection Algorithms in Protein–Protein Interaction for Drug Target Discovery and Drug Repurposing

**DOI:** 10.3389/fphar.2019.00109

**Published:** 2019-02-19

**Authors:** Jun Ma, Jenny Wang, Laleh Soltan Ghoraie, Xin Men, Benjamin Haibe-Kains, Penggao Dai

**Affiliations:** ^1^National Engineering Research Center for Miniaturized Detection Systems, Northwest University, Xi’an, China; ^2^Princess Margaret Cancer Centre, University Health Network, Toronto, ON, Canada; ^3^Shaanxi Microbiology Institute, Xi’an, China

**Keywords:** breast cancer, PPI network, target gene, drug repositioning, drug action, CMap, cell proliferation

## Abstract

The interactions between drugs and their target proteins induce altered expression of genes involved in complex intracellular networks. The properties of these functional network modules are critical for the identification of drug targets, for drug repurposing, and for understanding the underlying mode of action of the drug. The topological modules generated by a computational approach are defined as functional clusters. However, the functions inferred for these topological modules extracted from a large-scale molecular interaction network, such as a protein–protein interaction (PPI) network, could differ depending on different cluster detection algorithms. Moreover, the dynamic gene expression profiles among tissues or cell types causes differential functional interaction patterns between the molecular components. Thus, the connections in the PPI network should be modified by the transcriptomic landscape of specific cell lines before producing topological clusters. Here, we systematically investigated the clusters of a cell-based PPI network by using four cluster detection algorithms. We subsequently compared the performance of these algorithms for target gene prediction, which integrates gene perturbation data with the cell-based PPI network using two drug target prioritization methods, shortest path and diffusion correlation. In addition, we validated the proportion of perturbed genes in clusters by finding candidate anti-breast cancer drugs and confirming our predictions using literature evidence and cases in the ClinicalTrials.gov. Our results indicate that the Walktrap (CW) clustering algorithm achieved the best performance overall in our comparative study.

## Introduction

Drugs physically bind specific target proteins and activate downstream effectors to ultimately change the gene expression profiles of tumor cells, which are highly modular in the context of molecular interaction networks (Hartwell et al., 1999; Berger and Iyengar, 2009; Sah et al., 2014). Investigation of the modular properties of interactomes, such as protein–protein interaction (PPI) networks, can facilitate further discovery of the underlying molecular interaction mechanisms that drive cell response under specific conditions, such as drug treatment (Isik et al., 2015). Previous studies have used interaction networks to predict gene function, identify novel disease-related genes and to understand the overlapping association across disease phenotypes (Sharan et al., 2007; Lee et al., 2008; Menche et al., 2015). Recently, computational approaches have been used to build topological clusters as functional modules in PPI networks. For example, Spirin and Mirny identified modules in the PPI network through three methods and subsequently demonstrated the association between topological clusters and functional modules (Spirin and Mirny, 2003). Additionally, Kenley and Cho proposed a graph entropy algorithm to identify functional clusters from PPI networks (Kenley and Cho, 2011). These efforts have led to more effective modeling of PPIs and the drug targeting thereof with respect to specific diseases.

In the last decades, cancer cell lines have been widely used as models for understanding cancer biology and cellular response to anticancer drugs (Goodspeed et al., 2016). These cell lines have not only been comprehensively profiled at the molecular level, but they have also been used in large pharmacogenomic studies. The Connectivity Map (CMap) contains to date more than 7,000 expression profiles in five cancer cell lines (MCF7 and ssMCF7, human breast adenocarcinoma cell line; HL-60, human promyelocytic leukemia cell line; PC-3, human prostate cancer cell line; SK-MEL-5, melanoma cell line), screened for transcriptional responses induced by 1,309 small molecule compounds at varying concentrations from 6,100 microarray experiments conducted using the Affymetrix HT_HG_U133A array with 22,283 probesets (Lamb et al., 2006). Previous studies have also reported that the CMap data can be used to link transcriptional biomarkers to known mechanisms of drug action, such as the thioridazine inhibition of the phosphatidylinositol-3′-kinase (PI3K)/AKT pathway in ovarian cancer cells (Bae et al., 2011), allowing the identification of drug target proteins and facilitating the process of drug repurposing (Babcock et al., 2013; Iskar et al., 2013; Wu et al., 2013; Musa et al., 2017). In this study, we extensively used the transcriptomic and pharmacogenomic data pertaining to the cell lines found in the CMap to further understand the topological clustering in PPI networks from a comparative computational approach. We focused our interest on the MCF7 cell line, as it is the most commonly used cell line in human breast carcinoma, established in 1973 at the Michigan Cancer Foundation (Holliday and Speirs, 2011). Due to the hormone sensitivity found in MCF7 through the expression of the estrogen receptor (ER), this cell line has been reported as an ideal model to study hormone response in breast cancer (Levenson and Jordan, 1997).

**FIGURE 1:**
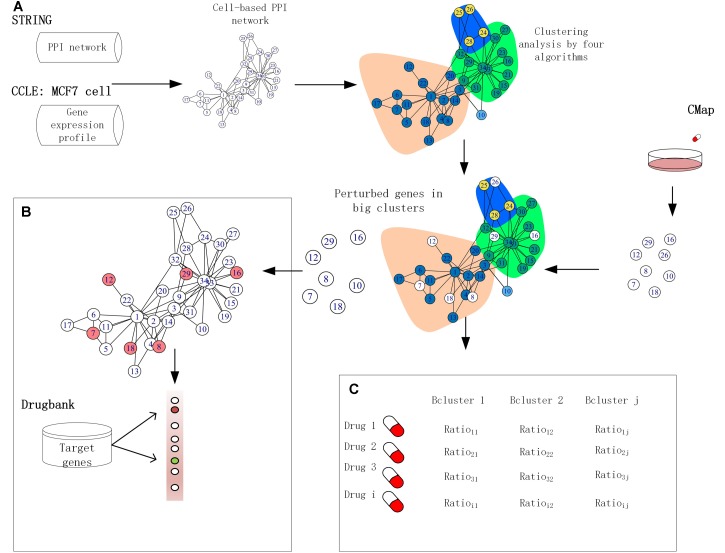
Framework for application of functional clusters. **(A)** The interactions in the human protein–protein interaction (PPI) network were removed if the corresponding proteins were not expressed in the MCF-7 cell lines. The clusters were built by four module detection algorithms. **(B)** The target genes were ranked based on the score produced by combining the perturbed genes of big size clusters with network information. **(C)** Cancer drugs by similarity analysis based on the fraction of perturbed genes in clusters.

Over the past few years, systems biology has made significant progress in addressing fundamental biological questions by making use of PPIs, leading to practical applications in drug target identification and drug discovery (Iskar et al., 2013; Wu et al., 2013; Ivliev et al., 2016). The STRING database (version 10.0) provides critical assessment and integrates all possible direct and indirect PPIs for more than 2,000 species, including 19,247 proteins with 8,548,002 interactions for *Homo sapiens* (Szklarczyk et al., 2015). Furthermore, various drug targeting measures have been developed, including a method called local radiality (LR) by Isik et al. (2015), integrating perturbed gene expression with PPI network information to prioritize drug target identification through different essential protein detection algorithms. The STRING database assigns a confidence score to each predicted protein–protein association calculated based on several sources, including published literature, experimental interaction data, and data concerning co-regulation of genes. However, despite these ongoing efforts that investigate cellular PPIs, due to the varying gene expression profiles in different cell lines, proteins exhibit dynamic behavior in interaction that current cell-agnostic PPI assignment methods do not fully recapitulate (Holliday and Speirs, 2011; Liang et al., 2014). As such, accuracy is poor for functional cluster prediction in individual cancer cell lines using existing clustering algorithms, and there remains a lack of convergence between the algorithms due to their diverse module detection theories and methods (Liu et al., 2017). Inspired by this, we developed a cell-based PPI network using the MCF7 cell line as an alternative to current cell-agnostic models. In this study, we compared the properties of functional clusters elucidated from a cell-based PPI network in the MCF-7 cell line produced by four module detection algorithms. We subsequently extract drug-induced functionally perturbed genes from the big clusters (defined as clusters with a size of greater than or equal to 10) detected by the algorithms and integrate them with MCF7 cell-based network information to improve the prioritization of target genes. Finally, we illustrate the potential association between perturbed genes and clusters in the MCF7 cell-based PPI network through investigations in drug repurposing. Our results highlight the validity of this comparative approach to identify novel anti-breast cancer drugs, which were further validated using literature and data from ClinicalTrials.gov (Figure 1). Furthermore, our results indicate that the Walktrap (CW) algorithm yields the best performance for detecting functional clusters in the PPI network.

## Materials and Methods

### Establishment of Cell-Based PPI Network Using MCF7

We downloaded the human PPIs from the STRING database (version 10) (Szklarczyk et al., 2015) to serve as the unfiltered PPIs for our cell-based PPI network. The ENSP IDs of the proteins found in STRING human PPI network were matched to their corresponding ENSG gene IDs using the R package biomaRt (version 2.34.2) (Badalà et al., 2009). To create the cell-based PPI network, we used as filtering criteria the gene expression data of the MCF7 cell line obtained from the Broad-Novartis Cancer Cell Line Encyclopedia (CCLE). The CCLE project provides public access to genomic data (Supplementary Table S1), as well as the analysis and visualization thereof for approximately 1,000 cancer cell lines (Barretina et al., 2012; Smirnov et al., 2018), with MCF7 being one of them. The gene expression data of the MCF7 cell line in CCLE were obtained using the R package PharmacoGx (version 1.12.0) (Smirnov et al., 2016), which contains the pharmacological profiles of several hundred cell lines generated by CCLE, the Genentech Cell Line Screening Initiative (gCSI) (Klijn et al., 2014), the Genomics of Drug Sensitivity in Cancer (GDSC) (Garnett et al., 2012) and the GRAY dataset, generated in Dr. Joe Gray’s lab at the Oregon Health and Science University (Daemen et al., 2013). To calculate the expression value of genes found in the MCF7 cell line, we converted the number of fragments per kilobase per million (FPKM) mapped reads units were converted to log2(FPKM+1) as the expression value of genes. We defined expressed genes is defined as those with a log2(FPKM+1) value of greater than or equal to 1 [log2(FPKM+1) ≥ 1] (Gonzalez-Porta et al., 2013). Using this definition, the MCF7 cell line expresses 13,096 genes. We then developed the cell-based PPI network subsetting the human PPI network to only include these expressed genes MCF7.

To limit non-existent and false-positive interactions between proteins in the MCF7 cell-based PPI network, we further filtered this network based on PPI confidence scores. The STRING repository has a confidence score for each PPI according to sources including existing literature, as well as co-expression and experimental data. A PPI has a high confidence score if there is supporting evidence for the interaction from a wide range of these sources. We selected PPIs with a confidence score of greater than 800 to be included in our final MCF7 cell-based network, which was also used in all downstream analysis. The MCF cell-based PPI network contains 7,904 protein members projecting 2,13,422 interactions, which represent the nodes and edges of the network, respectively.

### Functional Cluster Identification Algorithms

To date, a number of different criteria have been proposed for defining clusters in networks (Gao et al., 2009). A cluster is defined as a group of nodes in a network that are densely interconnected to each other, while sparsely connected to other the nodes of the same network. In this study, four widely used clustering algorithms were evaluated, compared, and categorized based on the methods applied for cluster identification (Orman et al., 2012) (Supplementary Table S2): These algorithms were selected due to their short runtimes determined in preliminary tests using the R package igraph (version 1.2.1) (Csardi and Nepusz, 2006).

The leading eigenvector (CLE) algorithm detects densely connected clusters in a network graph by calculating the leading non-negative eigenvector of the modularity matrix of the graph (Newman, 2006). Pons and Latapy have proposed a module detection hierarchical structure algorithm called the Walktrap (CW) for module detection, which is a hierarchical structure algorithm. They argued that short random walks tend to stay in the same cluster (Pons and Latapy, 2005). In the label propagation (CLP) algorithm, each node is initialized with a unique numeric label and chooses the label that is dominated by its neighbors during an iterative process. The CLP algorithm tends to gather densely connected nodes with the same label that comprise a cluster (Raghavan et al., 2007). Lastly, we also obtained topological gene clusters by implementing the infomap (CI) algorithm, proposed by Rosvall and Bergstrom (2008), to decompose the cell-based PPI network into clusters by employing random walks to analyze the information flow through a network. The igraph R package igraph (version 1.2.1 number) (Csardi and Nepusz, 2006) was used to identify clusters in the cell-based PPI network using the four cluster detection algorithms with on default arguments. Afterwards, we extracted the big clusters produced by the four algorithms, defined as those with a size of greater than or equal to 10 members. We also defined small clusters as those with less than 10 members. The big clusters detected by each algorithm were considered as functional clusters and subsequently used for target gene prediction and drug repurposing.

To elucidate the effect of network size for the clustering by CI and CW, we calculated the ratio of proteins in the big clusters to the total number of proteins in both the cell-agnostic PPI and the MCF7 cell-based PPI networks. The formula for this calculation is shown below:

(1) $$r=\frac{P_{\text{b}}}{P_{\text{t}}}$$

where *P*_b_ represents number of proteins in the big clusters, and *P*_t_ stands for the total number of proteins in each PPI network.

### Evaluation of Biological Processes

The biological processes in Gene Ontology (GO), which provide functions of genes and gene products determined by biological process (BP), molecular function (MF), and cellular component (CC), were downloaded from Molecular Signatures Database^1^. We enriched connected protein members of each big cluster in the cell-based PPI network to the GO terms to annotate protein function as well as to confirm the mechanisms of action of candidate anti-breast cancer drugs through hypergeometric tests using the R package piano (version 1.18.1) (Väremo et al., 2013). The biological process GO terms with false discovery rate (FDR) ≤ 0.05 were considered.

### Drug Perturbation Signatures in Cancer Cell Lines

To curate drug perturbation data, we first accessed the normalized gene expression in the MCF7 cell line from CMap via PharmacoGx (version 1.12.0) (Smirnov et al., 2016). We used the drugPerturbationSig function to identify genes whose expression is differentially expressed upon drug treatment, creating a signature representing gene expression changes induced by each drug. The algorithm of this function uses a linear regression model for the effect of drug concentration on gene expression in cell lines, while adding a term controlling for the batch effect in the CMap dataset:

(2) $$G=\beta_{0}+\beta_{\text{i}}C_{\text{i}}+\beta_{\text{t}}T+\beta_{\text{d}}D+\beta_{\text{b}}B$$

where *G* stands for gene expression, *C*_i_ indicates the concentration of a given compound, *T* denotes the type of cell line, *D* represents the duration of the experiment, and *B* represents the experimental batch, while βs are the regression coefficients. The significance of the association between a drug and a gene was estimated by the statistical significance of βi, which was calculated using an *F*-test to determine the improvement in fit after inclusion of the term. The function returns four values including the estimated coefficient for concentration, the *t*-statistic, the p-value and the FDR associated with that coefficient in a 3D array with genes and drugs. The *t*-statistic, carries information regarding the direction (up or down) of regulation of a given gene to identify perturbation in microarray experiments (Guo et al., 2006; Jeffery et al., 2006). The *t*-statistic returned by the lm function in R was calculated by following equation:

(3) $$t=\frac{\beta_{\text{i}}}{\mathrm{SE}}$$

where βi is the regression coefficient of the sample regression line, and SE is the standard error of the slope. After pre-processing, genes whose *p*-value was less than 0.01 were considered perturbed and their absolute *t*-statistic values were used as differential expression data. In CMap, the MFC7 cell line was treated with 1,255 drugs. Using the drugPerturbationSig function, we discovered in this cell line 11,833 distinct perturbed genes whose expression profiles were subsequently analyzed downstream this study.

### Integration of Gene Perturbation Data With Cell-Based PPI Network

We argue that perturbed genes participate in one of the big clusters generated by the clustering algorithms, and will be highly related to the mechanism of action of the drug that induced said perturbation. To integrate gene perturbation data into the MCF7 cell-based PPI network, we further filtered the network so that the perturbed genes were included only if they participate in the big clusters of each algorithm.

### Drug Target Prioritization in MCF7 Cell-Based PPI Network With Gene Perturbation Data

To date, several methods have been used to combine gene perturbation data with network information to find the target genes of compounds. Laenen et al. (2013) proposed a local measure called correlation diffusion (CD) that stands for a random walk-based algorithm to predict drug target genes. They first filtered out all connectivity correlation coefficients based on a certain t-threshold and normalized the coefficient value for each retaining correlation of its corresponding gene. Afterwards, they only considered single genes and their directly connected perturbed neighbors in the filtered network to compute the score for target gene prioritization. In contrast, a global measure using a shortest path (SP) approach combines the entire network topology propriety and perturbation data to calculate the gene score (Isik et al., 2015). The hypothesis for SP is that perturbed genes should have the shortest path to the target genes in the network. For this study, we used CD and SP to prioritize drug targets produced by the four clustering algorithms to compare the accuracy of each. We first revised the CD and SP methods to prioritize drug targets in the MCF7 cell-based network (CCD and CSP, respectively) with the perturbed genes, and the outcome of the evaluation of the four algorithms by the two methods are assigned new acronyms (CCD_CLE, CCD_CLP, CCD_CW, and CCD_CI; CSP_CLE, CSP_CLP, CSP_CW, and CSP_CI, respectively). Here, we show the revised formulations as follows for the two algorithms:

For CCD:

(4) $$M_{\text{ij}}=\{\begin{array}{l} \;\frac{P_{\text{ij}}}{\sum_{\text{j}}P_{\text{ij}}},\;i,j\;\in \;E_{\mathrm{cell}}\&P_{\text{ij}}>800\\ 0 \end{array}$$

where *P*_ij_ is the confidence value score of genes *i* and *j* expressed in the MCF7 cell-based PPI network. Normalizing the confidence scores based on the remaining connections per protein, the elements of a normalized interaction matrix *M* can be defined as formula 4. Multiplication of the *T*_pr_ with this matrix results in a ranking score for each gene:

(5) $$\begin{array}{cc} S=T_{\text{Pr}}\;M, & \mathrm{Pr}\in \;C_{\mathrm{size}\geq 10} \end{array}$$

where *T*_pr_ is a *t*-statistic value of each perturbed gene. The perturbed genes in CCD_CLE, CCD_CLP, CCD_CW, and CCD_CI are filtered as those found in the big clusters of each clustering algorithm. The ranking score of each gene in CCD is computed by integrating the MCF-7 cell-based PPI network with all Prs perturbed genes. The control group (CD_C) combine the PPI network (confidence score > 800) with all Prs perturbed genes induced by each drug.

For CSP:

(6) $$G_{\text{ij}}=\{\begin{array}{l} \;P_{\text{ij}},\;i,j\in E_{\mathrm{cell}}\&P_{\text{ij}}>800\\ 0 \end{array}$$

(7) $$S=\sum_{\mathrm{Pr}\in C_{\mathrm{size}\geq 10}}\text{sp}(n,\mathrm{Pr},G),\;n\in G$$

where *P*_ij_ is the confidence score of genes *i* and *j* expressed in the MCF7 cell-based PPI network. *Pr* is defined as a perturbed gene of MCF-7 to a drug, and it belongs to a big cluster as determined by the four algorithms. Lastly, we sort genes based on the score in increasing order for the CSP algorithm. Prioritized genes in CSP_CLE, CSP_CLP, CSP_CW, and CSP_CI were produced by the shortest path algorithm based on the functional perturbed gene and confidence score in MCF-7 cell-based PPI network. Gene’s scores in CSP group is calculate by shortest path between gene in MCF-7 cell-based PPI network and all perturbed gene induced by drug. We also computed the distance between genes in PPI network only filtered by confidence scores and all perturbed genes as control (SP_C).

### Drug Target Identification and Selection of Candidate Drugs

The target genes of the drugs used to treat the cell lines in the CMap database were downloaded from Drugbank ^2^ (Wishart et al., 2008), a database that comprehensively combines information about drug targets with that of drug action and that has been widely used for drug target discovery, drug design, and drug interaction prediction. We used Drugbank to determine target genes of drugs in the MCF7 cell-based PPI network. In the Drugbank database, 3,291 proteins are marked as the targets of approximately 4,900 drugs, 60% of which are including Food and Drug Administration (FDA)-approved drugs and tagged 10% are drugs under investigation (labeled as “experimental drugs”). The gene symbols were obtained by matching the UniProt identifier of the target genes in the drug target identifiers file ^3^. The UniProt database contains structure and function information of completely sequenced proteins, annotated with unique and stable identifiers (Consortium, 2017). After the mapping and filtering steps, 298 drugs with 270 target proteins were identified for the treated MCF-7 cell lines in CMap. This information was subsequently used to evaluate the performance of drug target prediction.

### Evaluation of Performance of Drug Prioritization

We evaluated the performance of the two target prioritization algorithms using precision-recall curves (PRC). The true positive (TP), true negative (TN), false positive (FP), and FN (false negative (FN) predictions were defined as previously described (Laenen et al., 2013). We divided the predictions into true and false sets based on each cut-off. TPs are all correctly predicted known targets above or equal to the rank cut-off. FPs are all proteins ranked above the cut-off, which are not in the known target set. FNs are known drug targets that are ranked below the cut-off and all remaining proteins are defined as TNs. The recall indicator is defined as

(8) $$\mathrm{Recall}=\frac{\mathrm{TP}}{\mathrm{TP}+\mathrm{FN}}$$

whereas the precision indicator is defined as

(9) $$\mathrm{Precision}=\frac{\mathrm{TP}}{\mathrm{TP}+\mathrm{FP}}$$

The two indicators were calculated using all possible thresholds from 1 to 13,184 (indicating the number of nodes in the MCF7 cell-based PPI network filtered by confidence scores) for the ranking list of drug targets. We made a PRC for the precision and recall of different rank cut-offs and calculated an area under the curve (AUC) value. Simple expression ranking can be set up as a baseline approach for performance assessment of the CCD and CSP network-based drug target ranking methods.

### Characteristic of Drugs in Clusters Across Four Detection Algorithms

We calculated the proportion of drug-perturbed genes in each big cluster across the four detection algorithms. The characteristic of a drug is evaluated by equation (10):

(10) $$\text{Ratio}=\frac{N_{C_{i}}}{N_{P}}$$

Where *N*_Ci_ denotes the number of perturbed genes within the *i*th big cluster, and *N*_P_ is the total number of drug-perturbed genes.

### Mechanism of Action of Candidate Breast Anticancer Drugs

For each drug, we extracted the perturbed genes of the drug within the biggest clusters to analyze function enrichments using hypergeometric tests. The biological process GO terms with an FDR of less than ≤ 0.05 were considered and overlapping biological processes were identified among candidate anti-breast cancer drugs. We generated the relationship of GO terms by Supek et al. (2011), which summarizes and visualizes long lists of GO terms. The Cytoscape tool (version 3.6.1) was used for GO term visualization.

## Results

### Clusters From the MCF7 Cell PPI Network

**FIGURE 2:**
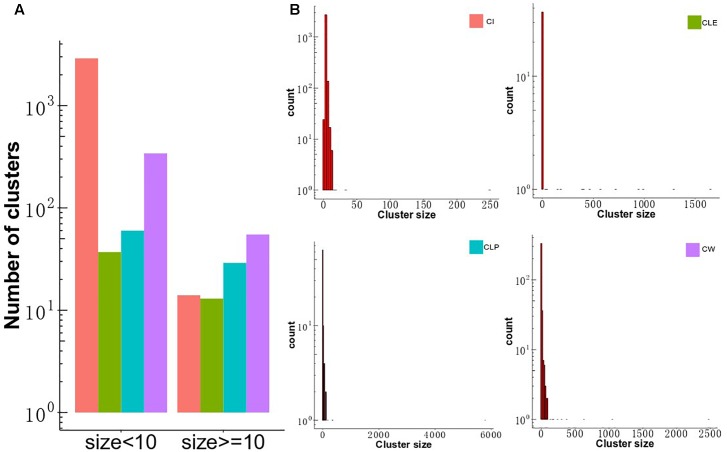
Distribution of cluster size and the overlap of the big clusters among the four algorithms. **(A)** The distribution of cluster size identified by CW, CLP, CLE, and CI algorithms in MCF-7 cell PPI network. The *y*-axis shows the number of clusters, and the *x*-axis gives two groups across four methods, which is classified based on the threshold of member number in each cluster. **(B)** Distribution of cluster size. The size of clusters detected by four different community partition methods (CLE, CLP, CW, and CI) in the MCF-7 cell PPI network. The *x*-axis represents the size of the cluster, and the *y*-axis describes the density of clusters.

To investigate differences in clustering structure produced by the four clustering algorithms, CLE, CLP, CW, and CI, we applied them to the MCF-7 cell-based PPI network, consisting of 7,904 proteins and 213,422 interactions. The four algorithms displayed a diversity of topological module sizes. We defined big clusters as those with a size of greater than or equal to 10 members and small clusters as those less than 10 members. The number of small clusters was larger than the number of big clusters in all algorithms (Figure 2A), which comprise a smaller proportion of the total number of clusters (15%, CW; 27.6%, CLE; 29%, CLP and; 0.3%, CI) (Supplementary Table S3), consistent with a previous report that compared the modular structure of human cell-agnostic PPI networks generated by seven community detection methods (Liu et al., 2017). The CW algorithm identified the largest number of big clusters (*n* = 55), as well as 341 small clusters. The CI algorithm detected 14 big clusters and 2,897 small clusters. Moreover, we calculated the ratio of the number of proteins in the clusters to the total number of proteins for all algorithms; the number of nodes was subsequently compared between (≥10 members) and small clusters (2 ≤ members < 10). Our results show that although the number of big clusters did not represent the majority of all clusters, the number of nodes included in big clusters of each algorithm, except CI, takes up the largest portion of the proteins in MCF7 cell-based PPI network. The distribution of cluster sizes is a crucial feature in determining the cluster structure in the four algorithms. It seems to follow a power-law distribution indicating that their sizes are heterogeneous, with many small clusters and only a few large ones (Figure 2B). For CLE, each cluster with a distinct size was detected only once, whereas the size distribution of the clusters detected by the other three algorithms had longer tails (Supplementary Figure S1).

**FIGURE 3:**
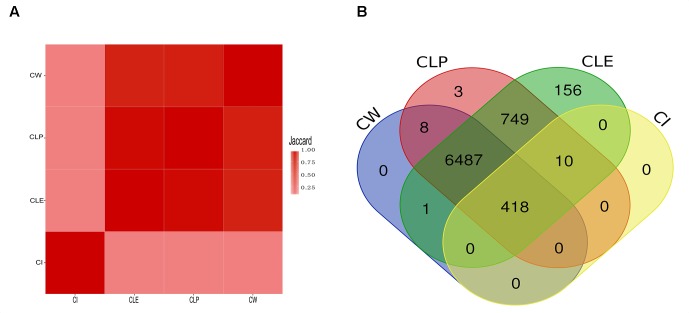
The similarity among the big clusters that were detected by four community detection methods. **(A)** The heatmap illustrates the Jaccard value of all big clusters of two algorithms. **(B)** The overlap of protein members that are clustered in big cluster among algorithms. All proteins in big clusters of CW and CI are shared with big clusters produced by CLE and CLP, while CLE and CLP contain its specific proteins in the big clusters.

To validate the agreement amongst the big clusters produced by the four algorithms, we computed the Jaccard index of protein members in all the big clusters between paired algorithms. We observed that most protein members in the big clusters identified by CW, CLP, and CLE are shared, as determined by high Jaccard indices (Figure 3A). We argue that the reason for this finding is that PPI networks naturally contain an underlying cluster structure and these three algorithms are hierarchical clustering methods. The CI algorithm is an exception because it identifies clusters based on coding theory and does not follow a hierarchical approach (Peel, 2010; Yang et al., 2016). Furthermore, clusters identified by CI require minimal bandwidth to represent some random walks in the network. We found that all four algorithms agree on 418 proteins in the big clusters. The CLP, CW, and CLE algorithms also share 6,487 proteins in their respective big clusters (Figure 3B). The big clusters generated by CLE contain a greater number of protein members (7,821 proteins) than other methods. On the other hand, 428 of 7,904 proteins are in the big clusters generated by CI. To elucidate the potential reason of cluster structure difference produced by CI, we further compared the number of protein members in the big clusters produced by CI with CW in the unfiltered cell-based PPI network (containing all connections) and filtered cell-based PPI network (confidence score > 800). CI is a compression-based approach, finding the clustering structure that provides the shortest description length for a random walk on the graph. Compared with the unfiltered cell-based PPI network, more proteins are grouped into big clusters in the filtered cell-based PPI network (Supplementary Figure S4A). Supplementary Figure S4B shows that the number of proteins in the filtered cell-based PPI network (confidence score > 800) plays an important role for clustering using the CI algorithm; more protein members can share the fixed connections to increase the median degree of each network (Supplementary Table S5), thus getting a greater number of nodes for big clusters. Thus, the CI algorithm considers most protein members with less degrees in the network as unimportant details and filters them out during clustering. Orman et al. (2011) also found that CI algorithm can produce a large number of small clusters from generated networks. Unlike the CI algorithm, the number of proteins and the number of connections has less impact for cluster size by the CW algorithm (Supplementary Figure S5).

### Go Term Analysis for Clusters

**FIGURE 4:**
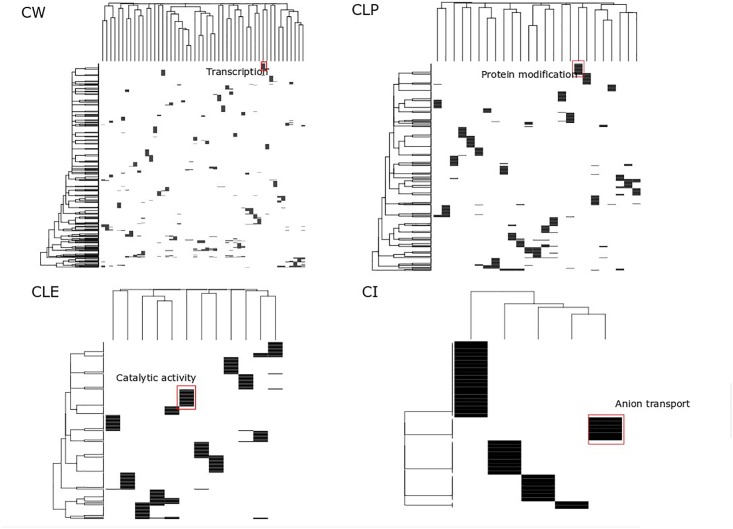
Functional analysis of big size clusters. This heatmap displays the top 20 significant associations between clusters and biological processes across four clusters detection algorithms. Color denotes enrichment of a given cluster with a biological process, hypergeometric log *p*-value after Benjamin–Hochberg adjustment. Cluster height reflects the number of interrelated processes associated with the given cluster. Cluster weight reflects clusters that shared this significant function. For major cluster based on each cluster detection algorithm, key biological themes are marked.

Clusters have been considered as function modules in the past decades (Qin and Gao, 2010). However, different clustering methods generate different protein clusters with different sizes and protein members, potentially confounding the function annotation for each cluster. For each algorithm, we searched for big clusters with shared biological processes, and we found that these big clusters were distinct when enriched to GO terms. This suggests that all proteins in the big clusters are essential not only in cancer cell growth but also participate in diverse biological processes, which are identified by different cluster detection approaches. In the heatmap shown in Figure 4, the rows represent the top 20 significant biological processes and the columns represent the clusters from the MCF7 cell-based PPI network. We found significantly distinct clusters in all of the four algorithms and highlighted the most significant biological processes in the biggest cluster in each of the four algorithms. For example, in the CW heatmap (Figure 4), the annotation “Transcription” denotes the biggest cluster determined by this algorithm and is associated with the regulation of transcription through the RNA polymerase II promoter. In addition, Supplementary Table S4 shows the adjusted *p*-values of these processes associated with the clusters. Approximately 3,000 biological processes of the big clusters are shared among CW, CLP, and CLE (Supplementary Figure S2). Furthermore, 51 of the 55 big clusters detected by CW contain biological processes that map to GO terms (Supplementary Figure S3), while a smaller number of big clusters in CLP, CLE, and CI (25, 12, and 5) is seen containing such processes. These results indicate that CLE, CLP, and CI can identify more complex functional clusters than CW.

### Cluster Improved Shortest Path Algorithm Performance

**FIGURE 5:**
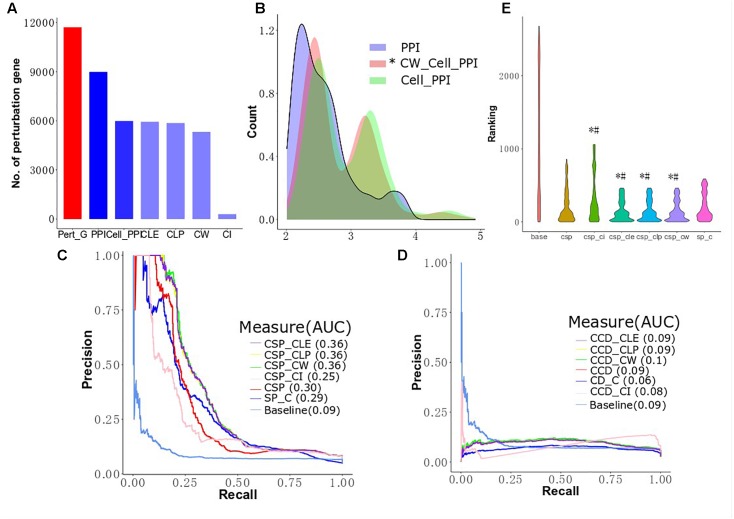
Cluster improving the performance of target prioritization. **(A)** The number of perturbed genes in MCF-7 cell lines after filtering by differential conditions. **(B)** Differentially expressed genes in clusters of cell PPI network are closer to known targets. The distributions of CW cell PPI are statistically different with PPI and cell PPI (Mann–Whitney, *p*-value < 2.2e^−16^ and *p*-value = 3.804e^−05^, respectively). **(C)** Performance of target gene prioritization using local radiality among functional genes selection algorithms (CLE, CLP, CW, and CI). **(D)** Performance of target gene prioritization using correlation diffusion among functional genes selection algorithms (CLE, CLP, CW, and CI). **(E)** The predictions are given for the top 200 of the target gene ranking lists. # means the target ranking curve is significant different with the curve of human network based on Mann–Whitney test; ^∗^ means the target ranking curve is significant different with the curve of cell-based network based on Mann–Whitney test.

Isik et al. (2015) integrated perturbed genes from drug-treated cell lines and human PPI networks to identify drug target genes. However, the expression profiles of genes in different cell lines are different and may influence the interaction between proteins (Liang et al., 2014). To improve the performance of drug target identification, we first selected genes exhibiting drug-induced perturbation signatures from the big clusters produced by the four algorithms and considered these genes as functionally perturbed genes (FPGs) of the drugs. Then, we focused on integrating the FPGs of each of the 298s mapped to Drugbank with the MCF-7 cell-based PPI network using our revised CSP and CCD algorithms to combine perturbed genes with MCF-7 cell-based PPI networks to prioritize target genes and calculate the AUC to compare the performance of several conditions. We prioritized 270 target genes for the 298 drugs using the ranks produced by the two algorithms, which were subsequently sorted to predict the possible targets of a given drug. Figure 5A shows the number of perturbed genes of the drugs in the network after several different filtering conditions. We observed 11,715 perturbed genes for the 298 drugs in MCF-7 before the application of any PPI networks. After integration of the human cell-agnostic PPI network from STRING, 8,987 perturbed genes were found, while 5,989 of these genes remained once the human PPI network was further filtered to control for the MCF7 cell line (i.e., the MCF7 cell-based network). We found that 5,321 functionally perturbed genes appeared in both MCF-7 cell-based PPI network and big clusters produced by CW algorithm. We found that removing non-existing interactions in the network reduced the number of FPs while elucidating the shortest distance from the target genes to the perturbed genes associated with individual drugs. We validate our comparative and integrative approach as we found that using perturbed genes found only in functional clusters for calculating the shortest distance proves more effective than using all perturbed genes. For both CD and SP, we integrated the cell-agnostic PPI network (with confidence score > 800) with all drug-induced perturbed genes, as opposed to FPGs, as control groups (SP_C and CD_C) (Figure 5B and Supplementary Figure S6). We observed in the controls that the data of the target genes and their corresponding drugs produced by CD are less than that generated by SP (Table 1). We found that the cell-based PPI network improved the performance of drug target ranking by first evaluating all perturbed genes without the integration of clusters (Figure 5C,D). The AUC value as determined by CSP for the cell-based group (CSP, 0.30) was higher than that of the cell-agnostic control (SP_C, 0.29). The CCD method found similar results: the AUC value of the cell-based group (CCD, 0.09) was also higher than that of the control group (CD_C, 0.06). For the CSP approach, perturbed proteins found in functional clusters (i.e., proteins translated from FPGs) can further improve target gene ranking; this outcome was not seen for CCD (Figure 5C,D). For instance, CSP_CLE, CSP_CLP, and CSP_CW have a higher AUC value compared to CSP (0.38 AUC). Figure 5D shows the results are similar for the AUC value across CLE, CLP, CW, and, CCD. The shape of the baseline curve is significantly different from all other curves. Collectively, these results indicated that network-based approaches demonstrate improved performance for drug target ranking, although the participation of perturbed proteins in functional clusters contribute less to drug target prioritization calculated by CCD.

**Table 1:** The 270 drug target genes extracted from MCF-7 cell-based PPI network filtered by *s* > 800.

	Number of target genes	Number of drugs
Cell_PPI	270	298
Cell_Shortest path	270	298
Cell_Diffusing correlation	163	163
Baseline	245	288

For the cell-based PPI network, the top 200 target genes rank in CLE, CLP, and CW, including 66 target genes shared between the three algorithms and corresponding to 127 drugs with significantly lower than CSP (Wilcoxon signed rank test, *p*-value = 0.04604, 0.04989, and 0.0347, respectively) and SP_C (Wilcoxon signed rank test, *p*-value = 0.000978, 0.001027, and 0.0006714, respectively) (Figure 5E). Top 200 target genes in baseline contains 102 unique target genes to 136 unique drugs. Most of the known targets and drugs in CW, CLP and CLE overlap with the top 200 genes in the ranking list of the CSP group (TCSP) (Table 2). These results show that the SP algorithm can further benefit the target gene ranking by integrating FPGs with network structure.

**Table 2:** The top 200 drug target genes in the ranking list.

	Target genes	Drugs	Overlapping target genes with TCSP	Overlapping drugs with TCSP
TCSP_CW	65	127	56	110
TCSP_CLP	66	127	57	110
TCSP_CLE	67	127	58	110
TCSP	72	127	72	127
TSP_C	65	123	44	96
TCI	87	119	54	83
Baseline	107	136	36	69

### Drug Repurposing in Breast Cancer Based on the Cluster

#### Comparing Potential Anti-cancer Drugs Identified by Cluster Detection Algorithms

**FIGURE 6:**
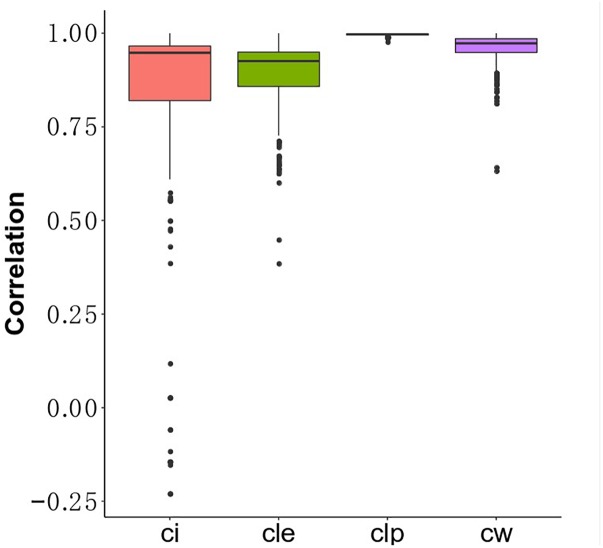
The distribution of Pearson value of fractions between drugs and tamoxifen.

Understanding the association between drugs and diseases at the molecular level is critical to unveil disease mechanisms and treatments (Zhao and Li, 2012). Drugs interact with targets and off-targets, inducing downstream pathway activity causing perturbations in the cellular transcriptome (Isik et al., 2015). The perturbed genes reflect the cellular response after drug treatment. If the perturbed genes of drugs that bind different target genes participate in the same functional clusters, these drugs could share a similar mode of action. Different algorithms generate different protein clusters with different sizes and protein members, potentially influencing functional annotation of each cluster. The hypothesis is that the fraction of perturbed genes of a drug in the clusters can reflect the properties of the drug. In this study, we used gene perturbation data integrated with clusters in the MCF7 cell-based network to find promising anti-breast cancer drugs. For each big cluster, we calculated the fraction of perturbed genes by each of the 298 drugs out of the total number of genes in that cluster, resulting in a matrix that illustrates the effect of each drug on each big cluster (Figure 1C). We then calculated the Pearson correlation of the perturbed gene proportions in the big clusters for the 298 drugs and compared it between these drugs and FDA-approved breast anticancer drugs, such as tamoxifen. Tamoxifen, an ER-targeting prodrug, is the most commonly administered chemotherapeutic drug in breast cancer patients. It is also the drug reported to induce the most gene perturbation in breast cancer cells. Figure 6 illustrates the distribution of the Pearson’s correlation between tamoxifen and the perturbed gene fractions. We found a strong correlation (*r* > 0.5) across CW, CLE, CLP, and CI. The reason for this result is that the bigger clusters of each algorithm usually include the most perturbed genes of drugs. We further predicted the top nine drugs from the drug ranking list for the each of the four algorithms as our candidate anti-breast cancer drugs, sorted based by the Pearson correlation coefficient. We demonstrated our predictions of each algorithm using evidence in the Clinical trials ^4^ and literature (Table 3). All top nine drugs predicted by CW demonstrated ability to inhibit cell growth or induce apoptosis in breast cancer cells, while the other three algorithms predicted fewer candidate anti-breast cancer drugs that fulfilled this aim. We found fulvestrant, geldanamycin, loperamide, and ouabain (an endogenous hormone) to be the common promising candidate anti-cancer drugs identified by both CW and CLP. Fulvestrant, a known ER antagonist, is recommended as a treatment option for ER-positive breast cancer (Lamb et al., 2006). Geldanamycin, a heat shock protein 90 inhibitor, has been evaluated for the cancer treatment in a phase III clinical trial (Shipp et al., 2011). Loperamide, a peripheral opiate agonist that induces a dose-dependent apoptosis and G2/M arrest in human cancer cell lines (Wen et al., 2012; Regan et al., 2014), is typically used as an antidiarrheal and was also identified as a promising anti-cancer drug by CI, in addition to CW and CLP. Furthermore, Raloxifene (identified by CLE), Fulvestrant (identified by CW and CLP), Geldanamycin (identified by CW and CLP), Tanespimycin (identified by CW), and Alvespimycin (identified by CLP) are currently in the process of being approved by clinical trials. Then, we performed drug repurposing on raloxifene, an FDA-approved breast anti-cancer drug, and for paclitaxel, an anti-mitotic anti-cancer agent, to identify their promising anti-cancer drugs, respectively. Three drugs chemically analogous to raloxifene detected by CW and CLP are currently being validated by clinical trials. Four raloxifene analogs identified by CW have *in vitro* evidence, while two discovered by CLP demonstrate cell experiment evidence (Supplementary Table S6). Supplementary Table S7 shows that four candidate drugs associated with paclitaxel detected by CW have been used on breast cancer patients in the Clinicaltrials database and two others drugs were proved *in vitro*. Therefore, integrating the clusters produced by the Walktrap algorithm with drug-induced differential gene expression yields the best performance for drug repurposing.

**Table 3:** Top nine potential anti-breast cancer drugs generated by the four algorithms.

	Drugs	Evidence	Indication in Drugbank
CW	FULVESTRANT	ClinicalTrials.gov	Anti-estrogen therapy
	TANESPIMYCIN	ClinicalTrials.gov	Treatment of leukemia (myeloid) and solid tumors
	GELDANAMYCIN	ClinicalTrials.gov	Not available
	LY294002	Hu et al., 2015	Not available
	TRIFLUOPERAZINE	Brosius et al., 2014	Anxiety disorders
	LOPERAMIDE	Gong et al., 2012	Acute non-specific diarrhea
	TROGLITAZONE	Yin et al., 2001	Antidiabetic and anti-inflammatory drug
	OUABAIN	Da Silva et al., 2014	Atrial fibrillation and flutter and heart failure
	CLOMIFENE	Murphy and Sutherland, 1983	Female infertility
CLE	HEXESTROL	None	Not available
	TROGLITAZONE	Yin et al., 2001	Antidiabetic and anti-inflammatory drug
	CHLORPROMAZINE	None	Schizophrenia
	PRENYLAMINE	None	Not available
	PREGNENOLONE	None	Not available
	RALOXIFENE	ClinicalTrials.gov	Osteoporosis
	MESTRANOL	None	Oral contraceptives
	PERPHENAZINE	None	Psychotic disorders
	OXYBENZONE	None	Sunscreen and other cosmetics
CLP	FULVESTRANT	ClinicalTrials.gov	Hormone receptor positive metastatic breast cancer
	LOPERAMIDE	Gong et al., 2012	Acute non-specific diarrhea
	PERHEXILINE	Ren et al., 2015	Severe angina pectoris
	OUABAIN	Da Silva et al., 2014	Atrial fibrillation and flutter and heart failure
	GELDANAMYCIN	ClinicalTrials.gov	Not available
	ALVESPIMYCIN	ClinicalTrials.gov	An antineoplastic agent for solid tumors
	MEFLOQUINE	Yan et al., 2013	Moderate acute malaria
	PERPHENAZINE	None	Psychotic disorders
	MIANSERIN	None	Depression
CI	PIMOZIDE	Zhou et al., 2016	Suppression of motor and phonic tics
	SULFASALAZINE	None	Crohn’s disease
	COLCHICINE	Sun et al., 2016	Acute gouty arthritis
	QUERCETIN	Duo et al., 2012	Not available
	PREGNENOLONE	None	Not available
	TRETINOIN	None	Remission in patients with acute promyelocytic leukemia (APL)
	MOMETASONE	None	Asthma
	LOPERAMIDE	Wen et al., 2012	Acute non-specific diarrhea
	FLUPHENAZINE	None	Psychotic disorders

#### Common Biological Processes of Candidate Breast Anti-cancer Drugs

**FIGURE 7:**
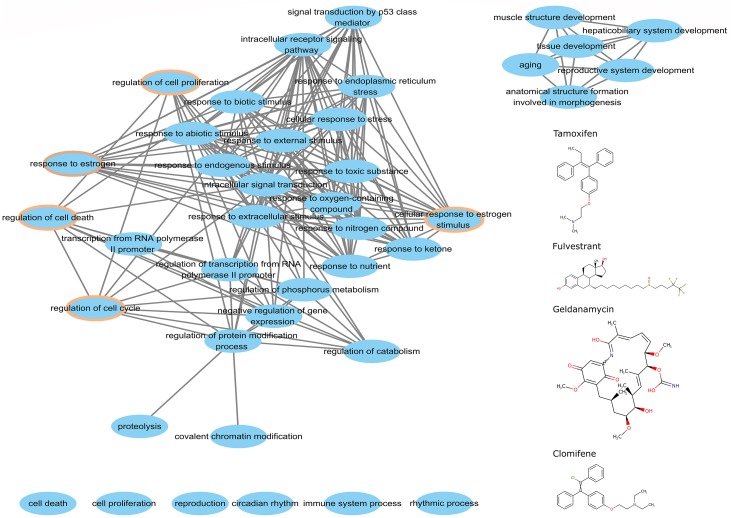
The common biological process of perturbed genes in the major cluster. The chemical structures of tamoxifen, fulvestrant, geldanamycin, and clomifene are on the right. The brown circle denotes the functional processes related to cell growth and development.

We argue that perturbed genes that participate big functional clusters are important for studying the mode of action of drugs. To better understand this association for the candidate anticancer drugs detected by the top-performing algorithm, we extracted the perturbed genes of tamoxifen, fulvestrant, geldanamycin, and clomifene within the biggest cluster of CW to analyze their functions using a hypergeometric test. We obtained the overlapping biological processes shared amongst the four drugs (Figure 7). We found that these drugs, despite their differences in chemical structure and target genes, share 33 common biological processes, 3 of which are directly involved in cell growth and development, including regulation of cell proliferation, cell death, and cell cycle. Moreover, the biological pathways associated with these drugs also shared the GO term for estrogen response (GO: 0043627).

## Discussion

The interactions among molecular components link biological functions, which are typically involved in functionally related clusters for their activities (Barabási and Oltvai, 2004; Liu et al., 2017). It has been reported that genes of similar disease phenotypes have a significantly higher number of interactions and highly connect with each other than with random genes (Goh and Choi, 2012), and the topological modules usually define disease-associated functional clusters (Ruan et al., 2006). However, Liu et al. (2017) pointed out the functional diversity of clusters in human PPI networks and that cluster detection approaches should be used with caution. Meanwhile, most studies classify the modules without considering the expression profile of specific cell lines or tissue types. In this study, we compared the clusters in the MCF-7 cell-based PPI network produced by four cluster detection algorithms, leading eigenvector (CLE), Walktrap [CWlabel propagation (CLP), and Infomap (CI)].

We observed that the big clusters (size ≥ 10) comprised a smaller fraction of total clusters and the big clusters generated by CLP, CLE, and CW contained most protein members, except CI. The distribution of node degrees in the network is a crucial factor for finding clusters using the CI algorithm. Therefore, we argue that compression-based clustering approaches should be used with caution for the identification of function clusters.

Drugs usually target multiple gene products and have the potential to be used for the treatment of several diseases. Some gene products may lead to serious side-effects and others may introduce novel applications to guide drug repurposing. Moreover, tumors are highly multifactorial at the molecular level, involving interactions in gene function networks (Zickenrott et al., 2017). The Connectivity Map (CMap) is a collection of gene expression profiles of five cancer cell lines before and after treatment with 1,300 small molecules (Lamb et al., 2006). In previous studies, integration of gene perturbation data in CMap with human PPI networks revealed drug targets and key pathways (Isik et al., 2015). However, most of the current network-based approaches for identification of essential proteins neglect significant differences in expression profiles among tissue types or even cell lines. We found that using a cell-based PPI network for target gene identification reduced the number of false positives for determining the shortest path between target and perturbed genes and improved drug target prediction compared with the cell-agnostic human PPI network. In this study, we improved target gene prediction by combining a cell-based PPI network with gene perturbation involved in big clusters.

Shortest path and correlation diffusion are two target gene prioritization algorithms. The SP measure assumes that deregulated genes might be close to drug targets based on network topology. SP identifies more diverse targets than CD, which agrees with previous conclusions (Isik et al., 2015). Moreover, we observed that integrating functional cluster-related perturbed genes with the MCF-7 cell-based network by CDhas little influence on the target gene prediction. The CD ranking, a random walk-based measure (Isik et al., 2015), sorts the proteins based on connectivity correlation in the network (Laenen et al., 2013). Cluster detection algorithms are also used as connection factor to identify functional clusters. Thus, the perturbed genes within the small clusters did not change the target gene ranking based on the this method. Furthermore, we calculated the fraction of drug-perturbed genes in each cluster as a drug-specific pattern to reflect cell response after drug treatment. We compared these patterns across tamoxifen, raloxifene, and paclitaxel, by computing the Pearson correlation to find promising anti-breast cancer candidate drugs. We observed that these drugs are highly similar to tamoxifen as predicted by CW, CLP, CLE, and CI. We selected the top nine drugs from each similarity ranking list as promising anti-breast cancer drugs and validated them through published literature and clinical trial cases. Based on the validation result, we found that the CW cluster detection algorithm showed the best ability to extract functional clusters from the network. To better understand the potential application of these drugs in breast cancer treatment, we performed a GO term analysis by extracting deregulated genes for four known anti-breast cancer drugs from the major cluster and obtained the overlapping biological processes among these drugs. The perturbed genes of these drugs not only appeared in big clusters detected from our cell-based PPI network, but also participated in common biological processes, such as cell cycle, cell death, and estrogen response pathways. We also used brinzolamide-induced gene perturbation data, which weakly correlates with tamoxifen, as a negative control, only three perturbed genes were found to overlap with gene members in the main cluster and no significant GO term enrichment was seen.

In summary, we gained insights into the structure of interactions by cluster detection algorithms and implemented the properties of clusters in the identification of target proteins and drug repurposing. We provide a new computational pipeline for the identification of drugs. However, the therapeutic potential of these agents requires further investigation.

## Author Contributions

JM, BH-K, and PD conceived and designed the study. JM and LG developed the methodology. JM, LG, JW, and XM analyzed and interpreted the data (e.g., statistical analysis, biostatistics, and computational analysis). JM, JW, BH-K, and XM wrote, reviewed, and/or revised the manuscript. BH-K and PD supervised the study.

## Conflict of Interest Statement

The authors declare that the research was conducted in the absence of any commercial or financial relationships that could be construed as a potential conflict of interest.

## Footnotes

1 http://software.broadinstitute.org/gsea/msigdb/index.jsp

2 www.drugbank.ca/releases/5-0-11/downloads/target-all-uniprot-links

3 www.drugbank.ca/releases/5-0-11/downloads/target-all-polypeptide-ids

4 www.clinicaltrials.gov

## References

[B1] Babcock J. J. Du F. Xu K. Wheelan S. J. Li M. (2013). Integrated analysis of drug-induced gene expression profiles predicts novel hERG inhibitors. *PLoS One* 8:e69513. 10.1371/journal.pone.0069513 23936032 PMC3720659

[B2] Badalà F. Nouri-mahdavi K. Raoof D. A. (2009). Mapping identifiers for the integration of genomic datasets with the R/Bioconductor package biomaRt. *Nat. Protoc.* 4 1184–1191. 10.1038/jid.2014.371 19617889 PMC3159387

[B3] Bae S. Kim B. Kang S. (2011). A gene signature-based approach identi fi es thioridazine as an inhibitor of phosphatidylinositol-3′ -kinase (PI3K)/AKT pathway in ovarian cancer cells. *Gynecol. Oncol.* 120 121–127. 10.1016/j.ygyno.2010.10.003 21035837

[B4] Barabási A.-L. Oltvai Z. N. (2004). Network biology: understanding the cell’s functional organization. *Nat. Rev. Genet.* 5 101–113. 10.1038/nrg1272 14735121

[B5] Barretina J. Caponigro G. Stransky N. Venkatesan K. Margolin A. A. Kim S. (2012). The cancer cell line encyclopedia enables predictive modelling of anticancer drug sensitivity. *Nature* 483:603. 10.1038/nature11003 22460905 PMC3320027

[B6] Berger S. I. Iyengar R. (2009). Network analyses in systems pharmacology. *Bioinformatics* 25 2466–2472. 10.1093/bioinformatics/btp465 19648136 PMC2752618

[B7] Brosius S. N. Turk A. N. Byer S. J. Longo J. F. Kappes J. C. Roth K. A. (2014). Combinatorial therapy with tamoxifen and trifluoperazine effectively inhibits malignant peripheral nerve sheath tumor growth by targeting complementary signaling cascades. *J. Neuropathol. Exp. Neurol.* 73 1078–1090. 10.1097/NEN.0000000000000126 25289889 PMC4458069

[B8] Castro L. S. Kviecinski M. R. Ourique F. Parisotto E. B. Grinevicius V. M. Correia J. F. (2016). Albendazole as a promising molecule for tumor control. *Redox Biol.* 10 90–99. 10.1016/j.redox.2016.09.013 27710854 PMC5053114

[B9] Consortium T. U. (2017). UniProt: the universal protein knowledgebase. *Nucleic Acids Res.* 45 158–169. 10.1093/nar/gkw1099 27899622 PMC5210571

[B10] Csardi G. Nepusz T. (2006). The igraph software package for complex network research. *InterJ. Complex Syst.* 1695 1–9.

[B11] Da Silva V. A. Da Silva K. A. Delou J. M. Da Fonseca L. M. Lopes A. G. Capella M. A. (2014). Modulation of ABCC1 and ABCG2 proteins by ouabain in human breast cancer cells. *Anticancer. Res.* 34 1441–1448. 24596392

[B12] Daemen A. Griffith O. L. Heiser L. M. Wang N. J. Enache O. M. Sanborn Z. (2013). Modeling precision treatment of breast cancer. *Genome Biol.* 14:R110. 10.1186/gb-2013-14-10-r110 24176112 PMC3937590

[B13] Deng X.-H. Song H.-Y. Zhou Y.-F. Yuan G.-Y. Zheng F.-J. (2013). Effects of quercetin on the proliferation of breast cancer cells and expression of survivin in vitro. *Exp. Ther. Med.* 6 1155–1158. 10.3892/etm.2013.1285 24223637 PMC3820718

[B14] Duo J. Ying G.-G. Wang G.-W. Zhang L. (2012). Quercetin inhibits human breast cancer cell proliferation and induces apoptosis via Bcl-2 and Bax regulation. *Mol. Med. Rep.* 5 1453–1456. 10.3892/mmr.2012.845 22447039

[B15] Gakhar G. Ohira T. Shi A. Hua D. H. Nguyen T. A. (2008). Antitumor effect of substituted quinolines in breast cancer cells. *Drug Dev. Res.* 69 526–534. 10.1002/ddr.20281

[B16] Gao L. Sun P. G. Song J. (2009). Functional Modules in Protein Interaction Networks. *J. Bioinform. Comput. Biol.* 7 217–242. 10.1142/S0219720009004023 19226668

[B17] Garnett M. J. Edelman E. J. Heidorn S. J. Greenman C. D. Dastur A. Lau K. W. (2012). Systematic identification of genomic markers of drug sensitivity in cancer cells. *Nature* 483 570–575. 10.1038/nature11005 22460902 PMC3349233

[B18] Goh K.-I. Choi I.-G. (2012). Exploring the human diseasome: the human disease network. *Brief. Funct. Genomics* 11 533–542. 10.1093/bfgp/els032 23063808

[B19] Gong X. W. Xu Y. H. Chen X. L. Wang Y. X. (2012). Loperamide, an antidiarrhea drug, has antitumor activity by inducing cell apoptosis. *Pharmacol. Res.* 65 372–378. 10.1016/j.phrs.2011.11.007 22119769

[B20] Gonzalez-Porta M. Frankish A. Rung J. Harrow J. Brazma A. (2013). Transcriptome analysis of human tissues and cell lines reveals one dominant transcript per gene. *Genome Biol.* 14:R70. 10.1186/gb-2013-14-7-r70 23815980 PMC4053754

[B21] Goodspeed A. Heiser L. M. Gray J. W. Costello J. C. (2016). Tumor-derived cell lines as molecular models of cancer pharmacogenomics. *Mol. Cancer Res.* 14 3–13. 10.1158/1541-7786.MCR-15-0189 26248648 PMC4828339

[B22] Guo L. Lobenhofer E. K. Wang C. Shippy R. Harris S. C. Zhang L. (2006). Rat toxicogenomic study reveals analytical consistency across microarray platforms. *Nat. Biotechnol.* 24 1162–1169. 10.1038/nbt1238 17061323

[B23] Hartwell L. H. Hopfield J. J. Leibler S. Murray A. W. (1999). From molecular to modular cell biology. *Nature* 402 C47–C52. 10.1038/35011540 10591225

[B24] Holliday D. L. Speirs V. (2011). Choosing the right cell line for breast cancer research. *Breast Cancer Res.* 13:215. 10.1186/bcr2889 21884641 PMC3236329

[B25] Hu Y. Guo R. Wei J. Zhou Y. Ji W. Liu J. (2015). Effects of PI3K inhibitor NVP-BKM120 on overcoming drug resistance and eliminating cancer stem cells in human breast cancer cells. *Cell Death Dis.* 6:e2020. 10.1038/cddis.2015.363 26673665 PMC4720896

[B26] Inchiosa M. A. Jr. (2018). Anti-tumor activity of phenoxybenzamine and its inhibition of histone deacetylases. *PLoS One* 13:e0198514. 10.1371/journal.pone.0198514 29897996 PMC5999115

[B27] Isik Z. Baldow C. Cannistraci C. V. Schroeder M. (2015). Drug target prioritization by perturbed gene expression and network information. *Sci. Rep.* 5:17417. 10.1038/srep17417 26615774 PMC4663505

[B28] Iskar M. Zeller G. Blattmann P. Campillos M. Kuhn M. Kaminska K. H. (2013). Characterization of drug-induced transcriptional modules: towards drug repositioning and functional understanding. *Mol. Syst. Biol.* 9:662. 10.1038/msb.2013.20 23632384 PMC3658274

[B29] Ivliev A. E. t Hoen P. A. Borisevich D. Nikolsky Y. Sergeeva M. G. (2016). Drug repositioning through systematic mining of gene coexpression networks in cancer. *PLoS One* 11:e0165059. 10.1371/journal.pone.0165059 27824868 PMC5100910

[B30] Jeffery I. B. Higgins D. G. Culhane A. C. (2006). Comparison and evaluation of methods for generating differentially expressed gene lists from microarray data. *BMC Bioinformatics* 7:359. 10.1186/1471-2105-7-359 16872483 PMC1544358

[B31] Juarez M. Schcolnik-Cabrera A. Dueñas-Gonzalez A. (2018). The multitargeted drug ivermectin: from an antiparasitic agent to a repositioned cancer drug. *Am. J. Cancer Res.* 8 317–331. 29511601 PMC5835698

[B32] Kenley E. C. Cho Y. R. (2011). *Detecting Protein Complexes and Functional Modules from Protein Interaction Networks: A Graph Entropy Approach.* Available at: http://onlinelibrary.wiley.com/doi/10.1002/pmic.201100193/full

[B33] Klijn C. Durinck S. Stawiski E. W. Haverty P. M. Jiang Z. Liu H. (2014). A comprehensive transcriptional portrait of human cancer cell lines. *Nat. Biotechnol.* 33 306–312. 10.1038/nbt.3080 25485619

[B34] Laenen G. Thorrez L. Börnigen D. Moreau Y. (2013). Finding the targets of a drug by integration of gene expression data with a protein interaction network. *Mol. Biosyst.* 9 1676–1685. 10.1039/c3mb25438k 23443074

[B35] Lamb J. Crawford E. D. Peck D. Modell J. W. Blat I. C. Wrobel M. J. (2006). The connectivity map: using gene-expression signatures to connect small molecules, genes, and disease. *Science* 313 1929–1935. 10.1126/science.1132939 17008526

[B36] Lee D.-S. Park J. Kay K. A. Christakis N. A. Oltvai Z. N. Barabási A.-L. (2008). The implications of human metabolic network topology for disease comorbidity. *Proc. Natl. Acad. Sci. U.S.A.* 105 9880–9885. 10.1073/pnas.0802208105 18599447 PMC2481357

[B37] Lee H. Kang S. Kim W. (2016). Drug repositioning for cancer therapy based on large-scale drug-induced transcriptional signatures. *PLoS One* 11:e0150460. 10.1371/journal.pone.0150460 26954019 PMC4783079

[B38] Levenson A. S. Jordan V. C. (1997). MCF-7: the first hormone-responsive breast cancer cell line. *Cancer Res.* 57 3071–3079. 9242427

[B39] Liang S.-S. Wang T.-N. Tsai E.-M. (2014). Analysis of protein-protein interactions in MCF-7 and MDA-MB-231 cell lines using phthalic acid chemical probes. *Int. J. Mol. Sci.* 15 20770–20788. 10.3390/ijms151120770 25402641 PMC4264195

[B40] Liu B. Huang X. Hu Y. Chen T. Peng B. Gao N. (2016). Ethacrynic acid improves the antitumor effects of irreversible epidermal growth factor receptor tyrosine kinase inhibitors in breast cancer. *Oncotarget* 7 58038–58050. 10.18632/oncotarget.10846 27487128 PMC5295410

[B41] Liu G. Wang H. Chu H. Yu J. Zhou X. (2017). Functional diversity of topological modules in human protein-protein interaction networks. *Sci. Rep.* 7:16199. 10.1038/s41598-017-16270-z 29170401 PMC5701033

[B42] Menche J. Sharma A. Kitsak M. Ghiassian S. D. Vidal M. Loscalzo J. (2015). Disease networks. Uncovering disease-disease relationships through the incomplete interactome. *Science* 347:1257601. 10.1126/science.1257601 25700523 PMC4435741

[B43] Murphy L. C. Sutherland R. L. (1983). Antitumor activity of clomiphene analogs in vitro: relationship to affinity for the estrogen receptor and another high affinity antiestrogen-binding site. *J. Clin. Endocrinol. Metab.* 57 373–379. 10.1210/jcem-57-2-373 6408114

[B44] Murren J. R. Durivage H. J. Buzaid A. C. Reiss M. Flynn S. D. Carter D. (1996). Trifluoperazine as a modulator of multidrug resistance in refractory breast cancer. *Cancer Chemother. Pharmacol.* 38 65–70. 10.1007/s002800050449 8603454

[B45] Musa A. Ghoraie L. S. Zhang S.-D. Galzko G. Yli-Harja O. Dehmer M. (2017). A review of connectivity map and computational approaches in pharmacogenomics. *Brief. Bioinform.* 19 506–523. 10.1093/bib/bbw112 28069634 PMC5952941

[B46] Narang V. S. Pauletti G. M. Gout P. W. Buckley D. J. Buckley A. R. (2007). Sulfasalazine-induced reduction of glutathione levels in breast cancer cells: enhancement of growth-inhibitory activity of Doxorubicin. *Chemotherapy* 53 210–217. 10.1159/000100812 17356269

[B47] Newman M. E. J. (2006). Finding community structure in networks using the eigenvectors of matrices. *Phys. Rev. E Stat. Nonlin. Soft Matter Phys.* 74:036104. 10.1103/PhysRevE.74.036104 17025705

[B48] Orman G. K. Labatut V. Cherifi H. (2011). Qualitative comparison of community detection algorithms. *Commun. Comput. Inf. Sci.* 167 265–279. 10.1007/978-3-642-22027-2-23 21945303

[B49] Orman G. K. Labatut V. Cherifi H. (2012). Comparative evaluation of community detection algorithms: a topological approach. *J. Stat. Mech. Theory Exp.* 2012:08001. 10.1088/1742-5468/2012/08/P08001 22202027

[B50] Pantziarka P. Bouche G. Meheus L. Sukhatme V. Sukhatme V. P. (2014). Repurposing Drugs in Oncology (ReDO)-mebendazole as an anti-cancer agent. *Ecancermedicalscience* 8:443. 10.3332/ecancer.2014.443 25075217 PMC4096024

[B51] Peel L. (2010). “Estimating network parameters for selecting community detection algorithms,” in *2010 13th International Conference on Information Fusion*, Piscataway, NJ, 1–8. 10.1109/ICIF.2010.5712065

[B52] Pons P. Latapy M. (2005). *Computing Communities in Large Networks Using Random Walks.* Available at: https://link.springer.com/chapter/10.1007/11569596_31

[B53] Pons P. Latapy M. (2006). Computing Communities in Large Networks Using Random Walks. *J. Graph Algorithms Appl.* 10 191–218. 10.7155/jgaa.00124

[B54] Qi C. Zhou Q. Li B. Yang Y. Cao L. Ye Y. (2014). Glipizide, an antidiabetic drug, suppresses tumor growth and metastasis by inhibiting angiogenesis. *Oncotarget* 5 9966–9979. 10.18632/oncotarget.2483 25294818 PMC4259451

[B55] Qin G. Gao L. (2010). Spectral clustering for detecting protein complexes in protein–protein interaction (PPI) networks. *Math. Comput. Model.* 52 2066–2074. 10.1016/j.mcm.2010.06.015

[B56] Raghavan U. N. Albert R. Kumara S. (2007). Near linear time algorithm to detect community structures in large-scale networks. *Phys. Rev.* E76:036106. 10.1103/PhysRevE.76.036106 17930305

[B57] Regan R. C. Gogal R. M. Barber J. P. Tuckfield R. C. Howerth E. W. Lawrence J. A. (2014). Cytotoxic effects of loperamide hydrochloride on canine cancer cells. *J. Vet. Med. Sci.* 76 1563–1568. 10.1292/jvms.13-0537 25649936 PMC4300369

[B58] Ren X.-R. Wang J. Osada T. Mook R. A. Jr. Morse M. A. Barak L. S. (2015). Perhexiline promotes HER3 ablation through receptor internalization and inhibits tumor growth. *Breast Cancer Res.* 17:20. 10.1186/s13058-015-0528-9 25849870 PMC4358700

[B59] Rosvall M. Bergstrom C. T. (2008). Maps of random walks on complex networks reveal community structure. *Proc. Natl. Acad. Sci. U.S.A.* 105 1118–1123. 10.1073/pnas.0706851105 18216267 PMC2234100

[B60] Ruan X.-G. Wang J.-L. Li J.-G. (2006). A network partition algorithm for mining gene functional modules of colon cancer from DNA microarray data. *Genomics Proteomics Bioinformatics* 4 245–252. 10.1016/S1672-0229(07)60005-9 17531800 PMC5054076

[B61] Sah P. Singh L. O. Clauset A. Bansal S. (2014). Exploring community structure in biological networks with random graphs. *BMC Bioinformatics* 15:220. 10.1186/1471-2105-15-220 24965130 PMC4094994

[B62] Sharan R. Ulitsky I. Shamir R. (2007). Network-based prediction of protein function. *Mol. Syst. Biol.* 3:88. 10.1038/msb4100129 17353930 PMC1847944

[B63] Shen T. Shang C. Zhou H. Luo Y. Barzegar M. Odaka Y. (2017). Ciclopirox inhibits cancer cell proliferation by suppression of Cdc25A. *Genes Cancer* 8 505–516. 10.18632/genesandcancer.135 28680535 PMC5489648

[B64] Shipp C. Watson K. Jones G. L. (2011). Associations of HSP90 client proteins in human breast cancer. *Anticancer. Res.* 31 2095–2101. 21737627

[B65] Smirnov P. Kofia V. Maru A. Freeman M. Ho C. El-Hachem N. (2018). PharmacoDB: an integrative database for mining in vitro anticancer drug screening studies. *Nucleic Acids Res.* 46 D994–D1002. 10.1093/nar/gkx911 30053271 PMC5753377

[B66] Smirnov P. Safikhani Z. El-Hachem N. Wang D. She A. Olsen C. (2016). PharmacoGx: an R package for analysis of large pharmacogenomic datasets. *Bioinformatics* 32 1244–1246. 10.1093/bioinformatics/btv723 26656004

[B67] Spirin V. Mirny L. A. (2003). Protein complexes and functional modules in molecular networks. *Proc. Natl. Acad. Sci. U.S A.* 100 12123–12128. 10.1073/pnas.2032324100 14517352 PMC218723

[B68] Sun Y. Lin X. Chang H. (2016). Proliferation inhibition and apoptosis of breast cancer MCF-7 cells under the influence of colchicine. *J. BUON* 21 570–575. 27569074

[B69] Supek F. Bošnjak M. Škunca N. Šmuc T. (2011). REVIGO summarizes and visualizes long lists of gene ontology terms. *PLoS One* 6:e21800. 10.1371/journal.pone.0021800 21789182 PMC3138752

[B70] Szklarczyk D. Franceschini A. Wyder S. Forslund K. Heller D. Huerta-Cepas J. (2015). STRING v10: protein-protein interaction networks, integrated over the tree of life. *Nucleic Acids Res.* 43 D447–D452. 10.1093/nar/gku1003 25352553 PMC4383874

[B71] Väremo L. Nielsen J. Nookaew I. (2013). Enriching the gene set analysis of genome-wide data by incorporating directionality of gene expression and combining statistical hypotheses and methods. *Nucleic Acids Res.* 41 4378–4391. 10.1093/nar/gkt111 23444143 PMC3632109

[B72] Wen X. Hong Y. Ling X. Xiang Y. (2012). Loperamide, an antidiarrhea drug, has antitumor activity by inducing cell apoptosis. *Pharmacol. Res.* 65 372–378. 10.1016/j.phrs.2011.11.007 22119769

[B73] Wishart D. S. Knox C. Guo A. C. Cheng D. Shrivastava S. Tzur D. (2008). DrugBank: a knowledgebase for drugs, drug actions and drug targets. *Nucleic Acids Res.* 36 D901–D906. 10.1093/nar/gkm958 18048412 PMC2238889

[B74] Wu Z. Wang Y. Chen L. (2013). Network-based drug repositioning. *Mol. Biosyst.* 9 1268–1281. 10.1039/c3mb25382a 23493874

[B75] Yan K.-H. Lin Y.-W. Hsiao C.-H. Wen Y.-C. Lin K.-H. Liu C.-C. (2013). Mefloquine induces cell death in prostate cancer cells and provides a potential novel treatment strategy in vivo. *Oncol. Lett.* 5 1567–1571. 10.3892/ol.2013.1259 23759954 PMC3678863

[B76] Yang Z. Algesheimer R. Tessone C. J. (2016). A comparative analysis of community detection algorithms on artificial networks. *Sci. Rep.* 6:30750. 10.1038/srep30750 27476470 PMC4967864

[B77] Yde C. W. Clausen M. P. Bennetzen M. V. Lykkesfeldt A. E. Mouritsen O. G. Guerra B. (2009). The antipsychotic drug chlorpromazine enhances the cytotoxic effect of tamoxifen in tamoxifen-sensitive and tamoxifen-resistant human breast cancer cells. *Anticancer. Drugs* 20 723–735. 10.1097/CAD.0b013e32832ec041 19584708

[B78] Yin F. Wakino S. Liu Z. Kim S. Hsueh W. A. Collins A. R. (2001). Troglitazone inhibits growth of MCF-7 breast carcinoma cells by targeting G1 cell cycle regulators. *Biochem. Biophys. Res. Commun.* 286 916–922. 10.1006/bbrc.2001.5491 11527386

[B79] Zhao S. Li S. (2012). A co-module approach for elucidating drug-disease associations and revealing their molecular basis. *Bioinformatics* 28 955–961. 10.1093/bioinformatics/bts057 22285830

[B80] Zheng H.-X. Wu L.-N. Xiao H. Du Q. Liang J.-F. (2014). Inhibitory effects of dobutamine on human gastric adenocarcinoma. *World J. Gastroenterol.* 20 17092–17099. 10.3748/wjg.v20.i45.17092 25493021 PMC4258577

[B81] Zhou W. Chen M.-K. Yu H.-T. Zhong Z.-H. Cai N. Chen G.-Z. (2016). The antipsychotic drug pimozide inhibits cell growth in prostate cancer through suppression of STAT3 activation. *Int. J. Oncol.* 48 322–328. 10.3892/ijo.2015.3229 26549437

[B82] Zickenrott S. Angarica V. E. Upadhyaya B. B. (2017). Prediction of disease–gene–drug relationships following a differential network analysis. *Cell Death Dis.* 7:e2040. 10.1038/cddis.2015.393 26775695 PMC4816176

