# Supplementary material for: A Comparative Study of Cluster Detection Algorithms in Protein–Protein Interaction for Drug Target Discovery and Drug Repurposing
Source: Front Pharmacol. 2019 Feb 19;10:109. doi: 10.3389/fphar.2019.00109 (PMC6389713; doi:10.3389/fphar.2019.00109)
Supplement: Supplementary file 1 [file Table_1.DOCX]

Table 1S. Data type in CCLE dataset

| Data Type | Assay/Platform | Raw | Processed |
| --- | --- | --- | --- |
| Pharmacological | CellTiter Glo | YES | AUC, IC50 |
| mRNA Expression | Affymetrix HG-U133PLUS2; | YES | BrainArray, Robust Multi-array Average (RMA) |
|  | Illumina RNA-seq | YES | No |
| DNA Copy Number | Affymetrix SNP 6.0 | YES | Birdseed, Normalized log2 ratios |
| DNA Mutation | OncoMap; | NO | Mutation Annotation Format (MAF) |
|  | Hybrid Capture | YES | MAF |

Table 2S. Clustering methods

| Methods | Algorithms | Category | Directed edges | Weighted edges |
| --- | --- | --- | --- | --- |
| cluster_walktrap (Pons and Latapy, 2006) | Computing communities in large networks using random walks | Node similarity-based approaches  Another | False | True |
| cluster_leading_eigen (Newman, 2006) | Finding community structure in networks using the eigenvectors of matrices | Modularity-based approaches | False | False |
| cluster_label_prop (Raghavan, U. N., Albert, R., Kumara, 2007) | Near linear time algorithm to detect community structures in large-scale networks. | Diffusion-based approaches | False | True |
| cluster_infomap (Rosvall and Bergstrom, 2008) | Minimal description length of a random walker using Huffman coding for each node | Compression-based approaches | True | True |

Table 3S. The number of clusters and the largest cluster size among four distinct algorithms.

| Methods | Number of clusters (10>Size≥2) | Number of big cluster (Size>10) | Percentage (#. big clusters/#. All clusters) | Largest cluster size |
| --- | --- | --- | --- | --- |
| CW | 286 | 55 | 15% | 2480 |
| CLE | 34 | 13 | 27.7% | 1643 |
| CLP | 60 | 29 | 29% | 5773 |
| CI | 2873 | 14 | 0.3% | 248 |

Table 5S. Distribution of degrees in the networks including fixed connections (213,422)

| #. proteins in Networks | Min. | 1^st^ Qu. | Median | Mean | 3^rd^ Qu. | Max. |
| --- | --- | --- | --- | --- | --- | --- |
| 7,904 | 1 | 3 | 11 | 27 | 30 | 2,883 |
| 8,726 | 1 | 3 | 11 | 25.02 | 29 | 3,003 |
| 10,446 | 1 | 6 | 13 | 20 | 29 | 499 |

Table 6S Highest similarity drugs for raloxifene among CW, CLE, CLP and CI.

| Methods | Drugs | Evidence | Indication in Drugbank |
| --- | --- | --- | --- |
| CW, CLE, CLP, CI | TAMOXIFEN | ClinicalTrials.gov | metastatic breast cancer |
| CW, CLE, CLP | MAPROTILINE | [(Lee et al., 2016)](https://paperpile.com/c/SKkscP/ucGk) | depression |
| CW, CLE | LOPERAMIDE | ClinicalTrials.gov | acute nonspecific diarrhea |
| CW, CLE | CHLORPROMAZINE | [(Yde et al., 2009)](https://paperpile.com/c/SKkscP/THCF) | schizophrenia |
| CLE, CLP | PERHEXILINE | [(Ren et al., 2015)](https://paperpile.com/c/SKkscP/aJXn). | severe angina pectoris |
| CW | FULVESTRANT | ClinicalTrials.gov | metastatic breast cancer |
| CW | TRIFLUOPERAZINE | [(Murren et al., 1996)](https://paperpile.com/c/SKkscP/sBzs) | anxiety disorders |
| CW | TROGLITAZONE | [(Yin et al., 2001)](https://paperpile.com/c/SKkscP/1k2s) | Type II diabetes mellitus |
| CLP | MIFEPRISTONE | ClinicalTrials.gov | medical termination of intrauterine pregnancy |
| CLP | TRIFLURIDINE | ClinicalTrials.gov | primay keratoconjunctivitis |
| CI | DOBUTAMINE | [(Zheng et al., 2014)](https://paperpile.com/c/SKkscP/2dkj) | cardiac decompensation |
| CI | ETACRYNIC ACID | [(Liu et al., 2016)](https://paperpile.com/c/SKkscP/ck7X) | high blood pressure |
| CW, CLE, CLP | CLOMIFENE | None | anovulation |
| CLE, CI | PERPHENAZINE | None | psychotic disorders |
| CW, CLP | HYDROFLUMETHIAZIDE | None | edema |
| CLE | DESIPRAMINE | None | endogenous depression |
| CLE | AMITRIPTYLINE | None | depressive disorder |
| CLP | DOSULEPIN | None | depressive illness |
| CLP | BRINZOLAMIDE | None | elevated intraocular pressure |
| CI | ALVESPIMYCIN | None | an antineoplastic agent |
| CI | ACETYLSALICYLIC ACID | None | relief of pain |
| CI | CHLORZOXAZONE | None | the relief of discomfort |
| CI | MEDRYSONE | None | allergic conjunctivitis |
| CI | ETOPOSIDE | None | refractory testicular tumors |

Table 7S Highest similarity drugs for paclitaxel among CW, CLE, CLP and CI.

| Methods | Drugs | Evidence | Indication in Drugbank |
| --- | --- | --- | --- |
| CW, CLE, CLP | CICLOPIROX | [(Shen et al., 2017)](https://paperpile.com/c/SKkscP/BrBd) | immunocompetent patients |
| CW, CLE | ETOPOSIDE | None | refractory testicular tumors |
| CW, CLE | TRIFLURIDINE | ClinicalTrials.gov | primay keratoconjunctivitis |
| CW, CLE | RESVERATROL | ClinicalTrials.gov | Herpes labialis infections |
| CW, CLE | ALBENDAZOLE | [(Castro et al., 2016)](https://paperpile.com/c/SKkscP/GluA) | parenchymal neurocysticercosis |
| CW | MYCOPHENOLIC ACID | ClinicalTrials.gov | the prophylaxis of organ rejection |
| CW | METHOTREXATE | ClinicalTrials.gov | gestational choriocarcinoma |
| CW | FINASTERIDE | None | symptomatic benign prostatic hyperplasia |
| CW | 8AZAGUANINE | None | Not available |
| CLE | FENOFIBRATE | ClinicalTrials.gov | adjunctive therapy |
| CLE | TRICHLORMETHIAZIDE | None | oedema |
| CLE | FLUOCINONIDE | None | anti-inflammatory |
| CLE | DIFLORASONE | None | relief of the inflammatory |
| CLP | SULFASALAZINE | [(Narang et al., 2007)](https://paperpile.com/c/SKkscP/P700) | Crohn's disease |
| CLP | QUERCETIN | [(Deng et al., 2013)](https://paperpile.com/c/SKkscP/RDvn) | Not Available |
| CLP | PHENOXYBENZAMINE | [(Inchiosa, 2018)](https://paperpile.com/c/SKkscP/OlAk) | phaeochromocytoma |
| CLP | MEBENDAZOLE | [(Pantziarka et al., 2014)](https://paperpile.com/c/SKkscP/iTsu) | Enterobius vermicularis |
| CLP | PRIMAQUINE | [(Gakhar et al., 2008)](https://paperpile.com/c/SKkscP/WT8H) | malaria |
| CLP | MEDRYSONE | None | allergic conjunctivitis |
| CLP | NORETHISTERONE | None | secondary amenorrhea |
| CLP | FENOPROFEN | None | relief of the signs and symptoms of rheumatoid arthritis |
| CI | PRENYLAMINE | None | Not Available |
| CI | IVERMECTIN | [(Juarez et al., 2018)](https://paperpile.com/c/SKkscP/8Tiq) | intestinal |
| CI | TOLBUTAMIDE | None | non-insulin-dependent diabetes mellitus |
| CI | PRIMIDONE | None | grand mal |
| CI | DORZOLAMIDE | None | elevated intraocular pressure |
| CI | VIGABATRIN | None | resistant epilepsy |
| CI | GLIPIZIDE | [(Qi et al., 2014)](https://paperpile.com/c/SKkscP/mSJd) | hyperglycemia |
| CI | TOLAZAMIDE | None | lower the blood glucose |
| CI | THEOBROMINE | None | vasodilator |


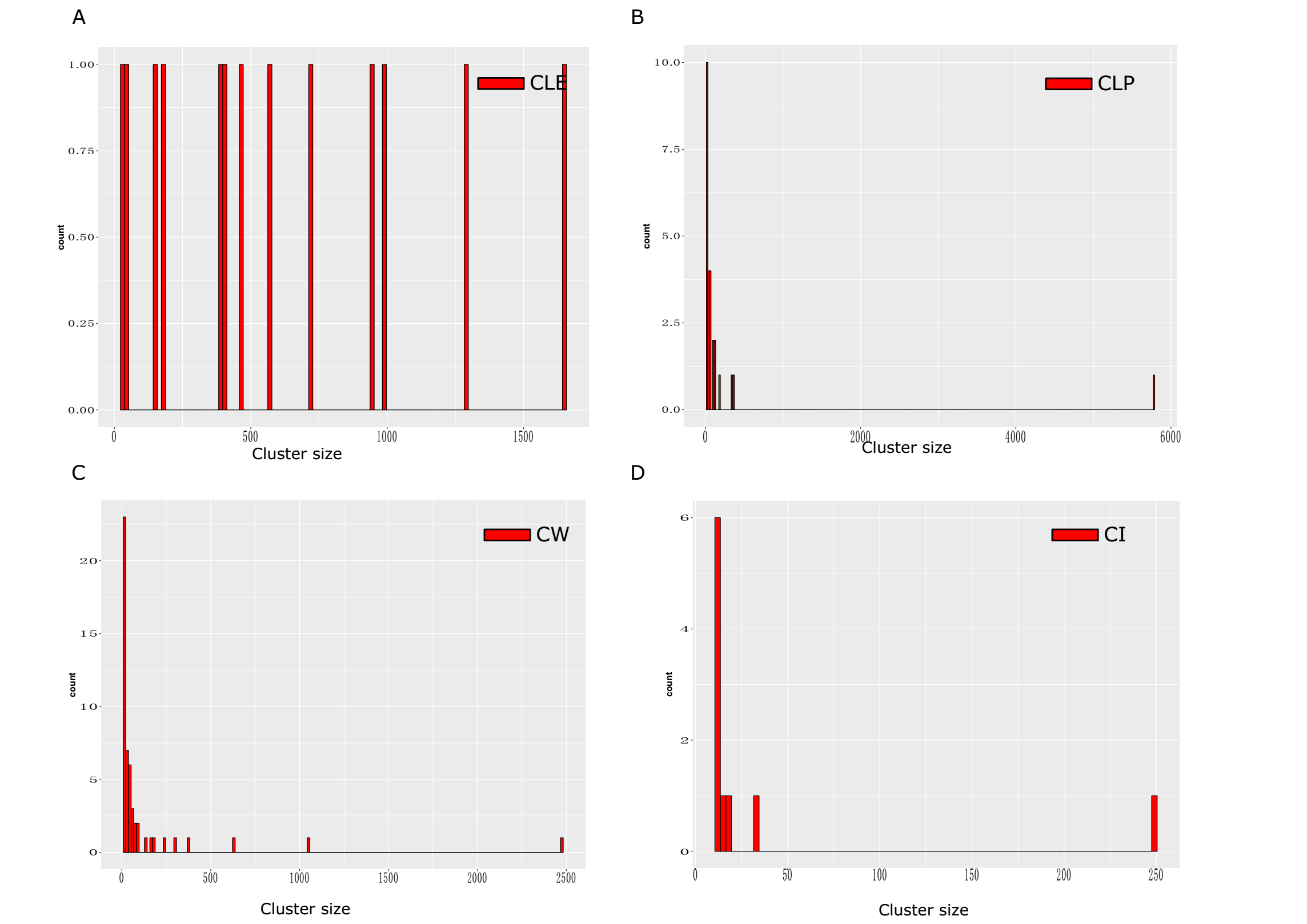


Figure 1S. Distribution of the big clusters among the four algorithms.


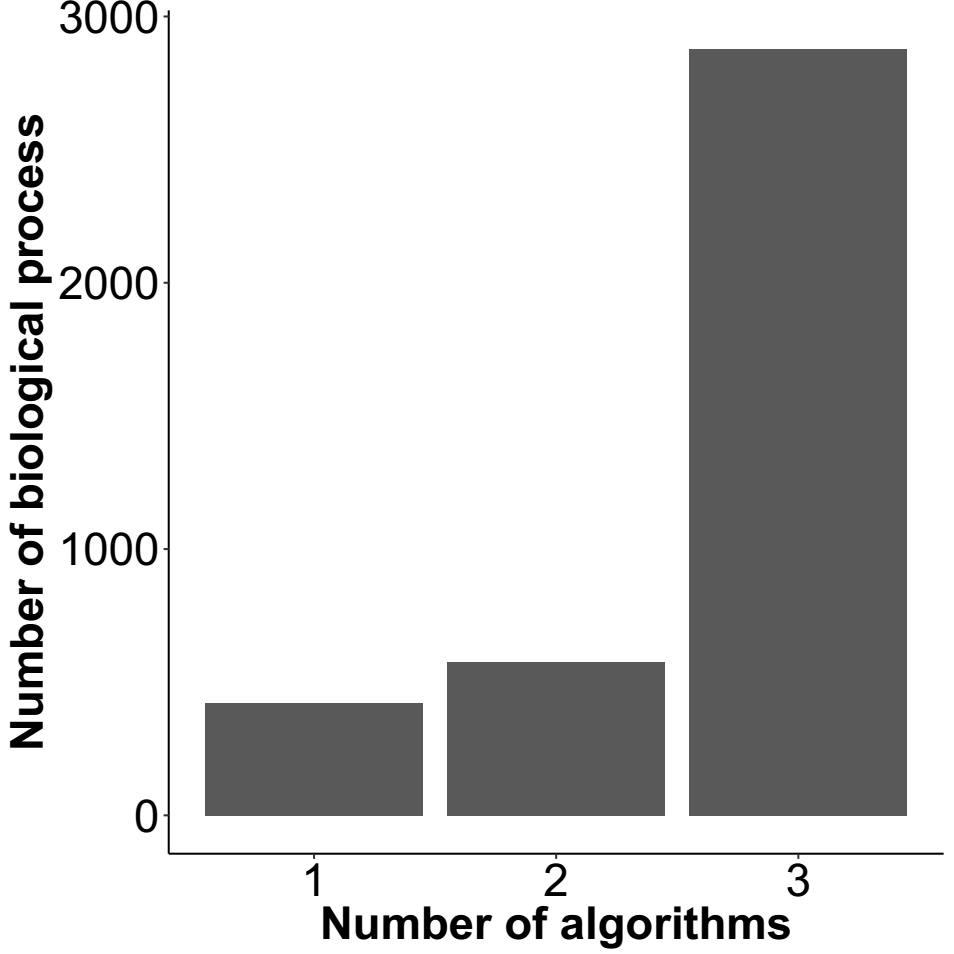


Figure 2S. The number of shared biological processes across CW, CLP and CLE algorithms.


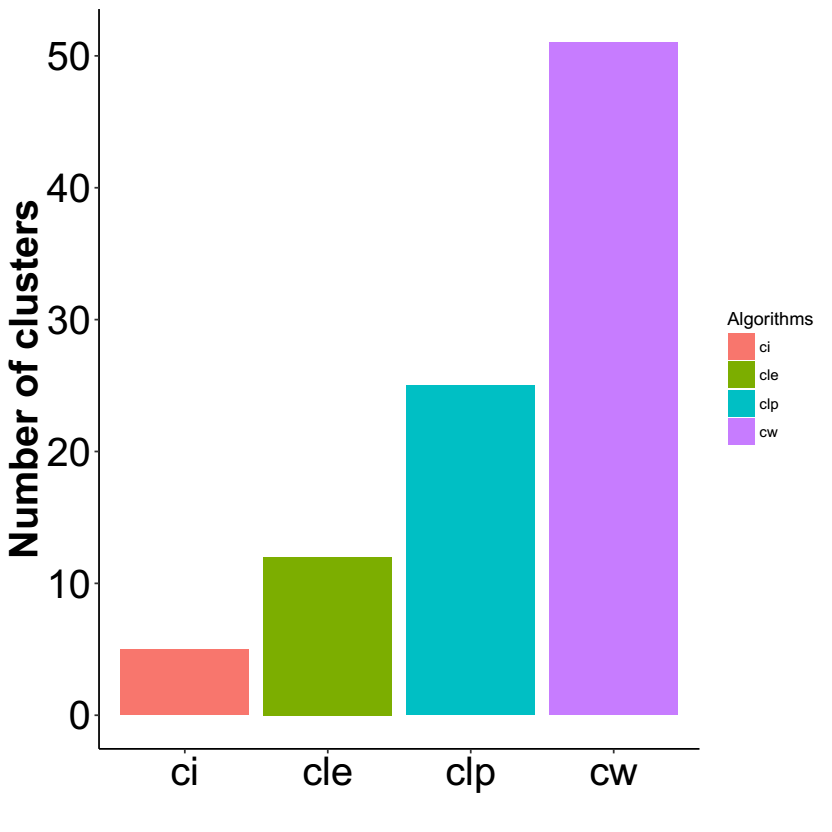


Figure 3S. The number of clusters containing biological processes among distinct algorithms.


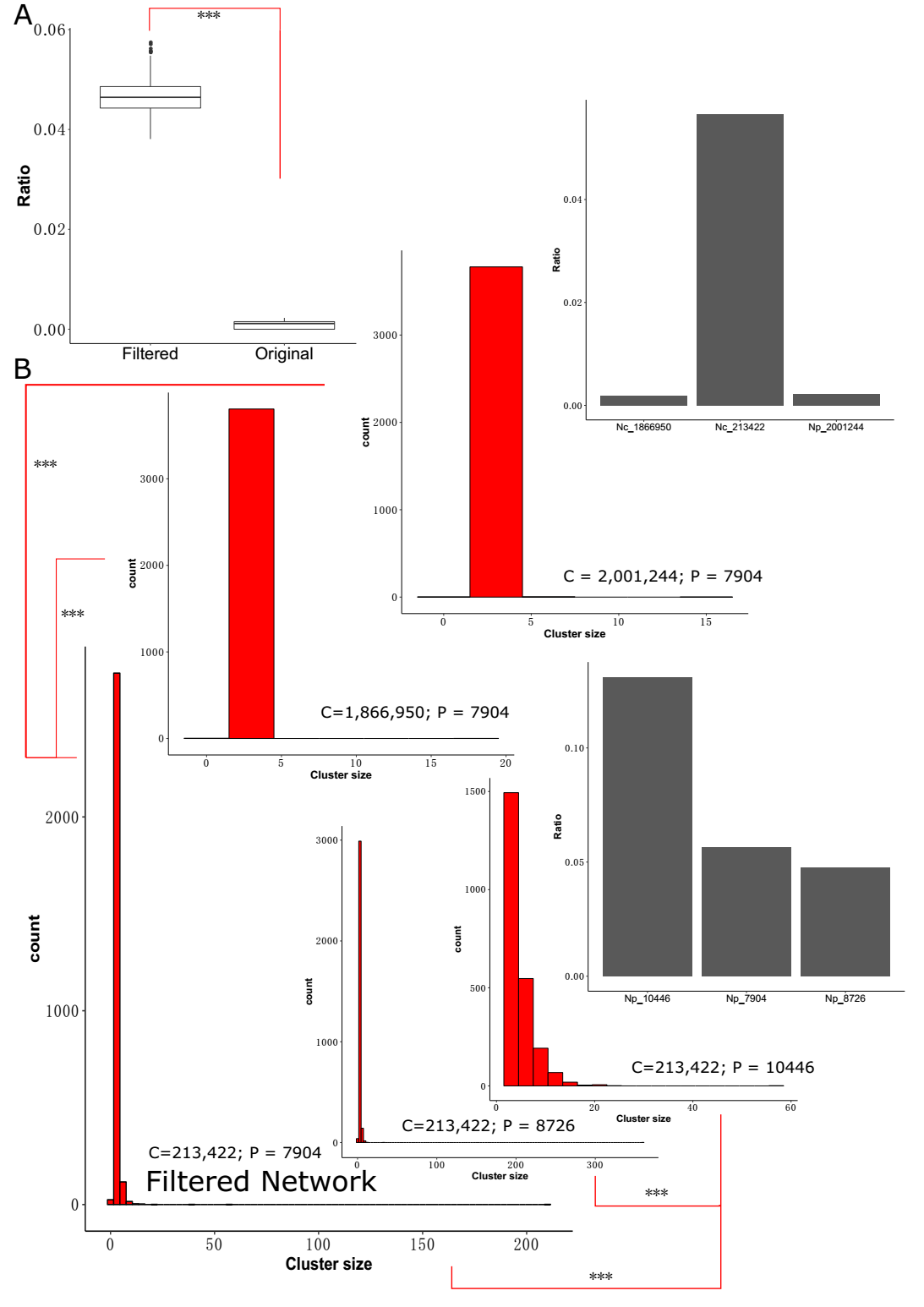


Figure 4S. Big clusters produced by CI algorithm based on vary networks.

**A** Ratio’s distribution in both two networks**. B** Distribution between cluster size based on different number of connections and nodes in filtered and original network. Bar plots mean ratio of each variable.


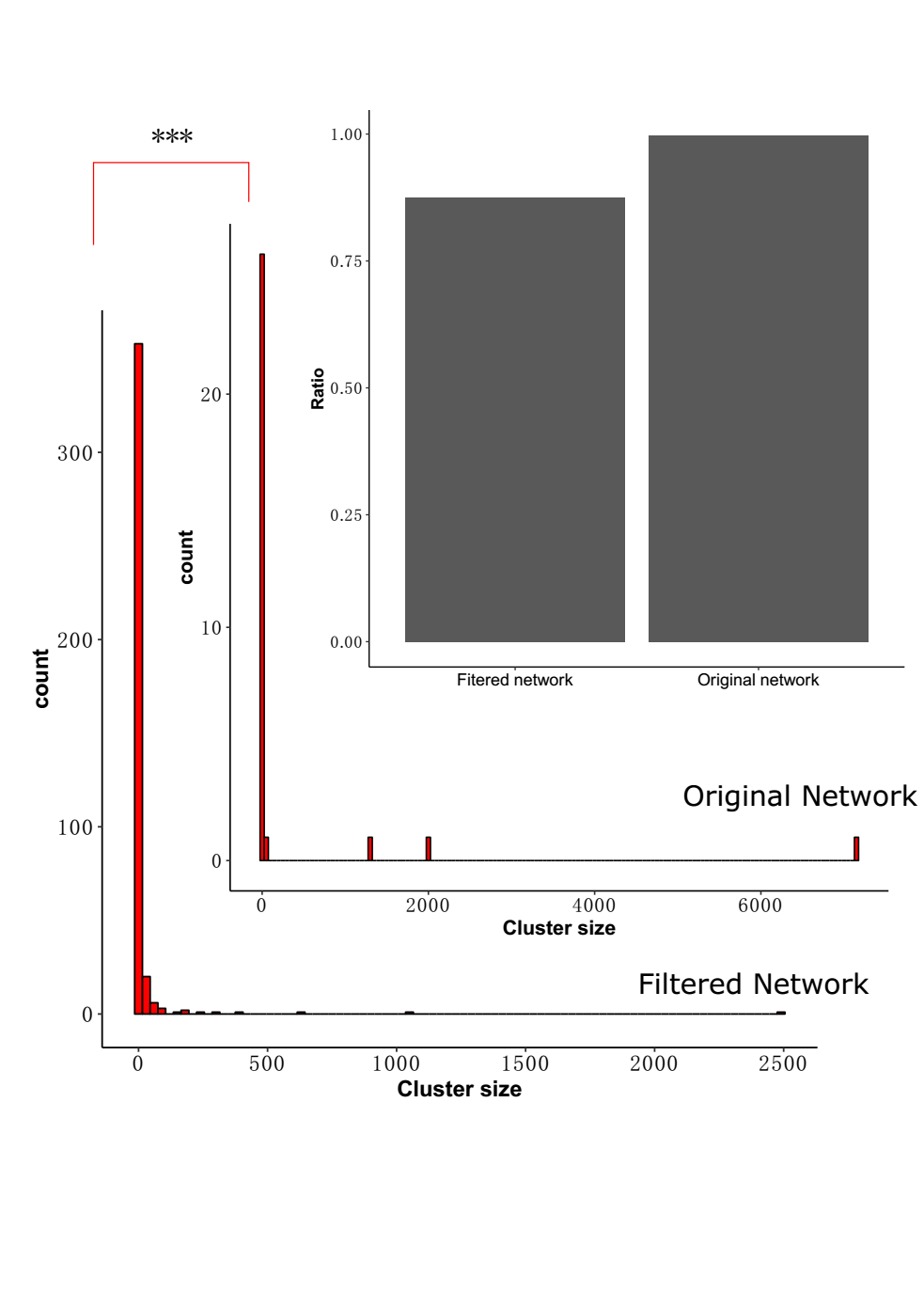


Figure 5S. Big clusters produced by CW algorithm based on two networks. The distribution of cluster size is significant different between original network and filtered network. Most proteins in two networks are cluster into big clusters and filtered network produces more big clusters than original network.


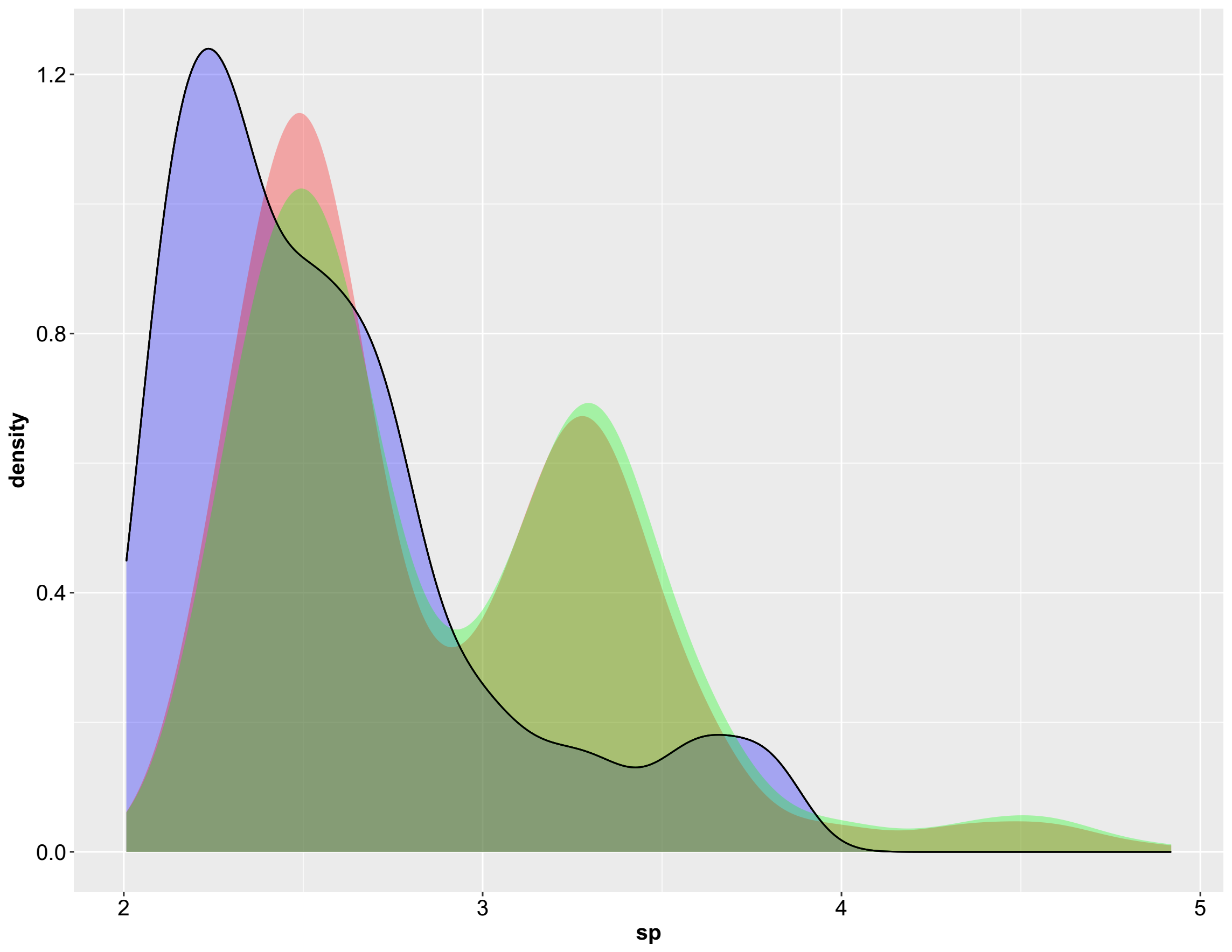


Figure 6S. Differentially expressed genes in clusters of Cell-based PPI network produced by CLP algorithm are closer to known targets. Blue means different PPI network with confidence score>800. Red and green represent distance calculation with/without clusters in Cell-based PPI network.

Newman, M. E. J. (2006). Finding community structure in networks using the eigenvectors of matrices. *Phys. Rev. E - Stat. Nonlinear, Soft Matter Phys.* 74. doi:10.1103/PhysRevE.74.036104.

Pons, P., and Latapy, M. (2006). Computing Communities in Large Networks Using Random Walks. *J. Graph Algorithms Appl.* 10, 191–218. doi:10.7155/jgaa.00124.

Raghavan, U. N., Albert, R., Kumara, S. (2007). *Near linear time algorithm to detect community structures in large-scale networks*. doi:10.4324/9780203440124.

Rosvall, M., and Bergstrom, C. T. (2008). Maps of random walks on complex networks reveal community structure. *Proc. Natl. Acad. Sci. U. S. A.* 105, 1118–1123.

[Castro, L. S. E. P. W., Kviecinski, M. R., Ourique, F., Parisotto, E. B., Grinevicius, V. M. A. S., Correia, J. F. G., et al. (2016). Albendazole as a promising molecule for tumor control. Redox Biol 10, 90–99.](http://paperpile.com/b/SKkscP/GluA)

[Deng, X.-H., Song, H.-Y., Zhou, Y.-F., Yuan, G.-Y., and Zheng, F.-J. (2013). Effects of quercetin on the proliferation of breast cancer cells and expression of survivin in vitro. Exp. Ther. Med. 6, 1155–1158.](http://paperpile.com/b/SKkscP/RDvn)

[Gakhar, G., Ohira, T., Shi, A., Hua, D. H., and Nguyen, T. A. (2008). Antitumor effect of substituted quinolines in breast cancer cells. Drug Dev. Res. 69, 526–534.](http://paperpile.com/b/SKkscP/WT8H)

[Hu, Y., Guo, R., Wei, J., Zhou, Y., Ji, W., Liu, J., et al. (2015). Effects of PI3K inhibitor NVP-BKM120 on overcoming drug resistance and eliminating cancer stem cells in human breast cancer cells. Cell Death Dis. 6, e2020.](http://paperpile.com/b/SKkscP/6wQK3)

[Inchiosa, M. A., Jr (2018). Anti-tumor activity of phenoxybenzamine and its inhibition of histone deacetylases. PLoS One 13, e0198514.](http://paperpile.com/b/SKkscP/OlAk)

[Juarez, M., Schcolnik-Cabrera, A., and Dueñas-Gonzalez, A. (2018). The multitargeted drug ivermectin: from an antiparasitic agent to a repositioned cancer drug. Am. J. Cancer Res. 8, 317–331.](http://paperpile.com/b/SKkscP/8Tiq)

[Lee, H., Kang, S., and Kim, W. (2016). Drug Repositioning for Cancer Therapy Based on Large-Scale Drug-Induced Transcriptional Signatures. PLoS One 11, e0150460.](http://paperpile.com/b/SKkscP/ucGk)

[Liu, B., Huang, X., Hu, Y., Chen, T., Peng, B., Gao, N., et al. (2016). Ethacrynic acid improves the antitumor effects of irreversible epidermal growth factor receptor tyrosine kinase inhibitors in breast cancer. Oncotarget 7, 58038–58050.](http://paperpile.com/b/SKkscP/ck7X)

[Murren, J. R., Durivage, H. J., Buzaid, A. C., Reiss, M., Flynn, S. D., Carter, D., et al. (1996). Trifluoperazine as a modulator of multidrug resistance in refractory breast cancer. Cancer Chemother. Pharmacol. 38, 65–70.](http://paperpile.com/b/SKkscP/sBzs)

[Narang, V. S., Pauletti, G. M., Gout, P. W., Buckley, D. J., and Buckley, A. R. (2007). Sulfasalazine-induced reduction of glutathione levels in breast cancer cells: enhancement of growth-inhibitory activity of Doxorubicin. Chemotherapy 53, 210–217.](http://paperpile.com/b/SKkscP/P700)

[Pantziarka, P., Bouche, G., Meheus, L., Sukhatme, V., and Sukhatme, V. P. (2014). Repurposing Drugs in Oncology (ReDO)-mebendazole as an anti-cancer agent. Ecancermedicalscience 8, 443.](http://paperpile.com/b/SKkscP/iTsu)

[Qi, C., Zhou, Q., Li, B., Yang, Y., Cao, L., Ye, Y., et al. (2014). Glipizide, an antidiabetic drug, suppresses tumor growth and metastasis by inhibiting angiogenesis. Oncotarget 5, 9966–9979.](http://paperpile.com/b/SKkscP/mSJd)

[Ren, X.-R., Wang, J., Osada, T., Mook, R. A., Jr, Morse, M. A., Barak, L. S., et al. (2015). Perhexiline promotes HER3 ablation through receptor internalization and inhibits tumor growth. Breast Cancer Res. 17, 20.](http://paperpile.com/b/SKkscP/aJXn)

[Shen, T., Shang, C., Zhou, H., Luo, Y., Barzegar, M., Odaka, Y., et al. (2017). Ciclopirox inhibits cancer cell proliferation by suppression of Cdc25A. Genes Cancer 8, 505–516.](http://paperpile.com/b/SKkscP/BrBd)

[Yde, C. W., Clausen, M. P., Bennetzen, M. V., Lykkesfeldt, A. E., Mouritsen, O. G., and Guerra, B. (2009). The antipsychotic drug chlorpromazine enhances the cytotoxic effect of tamoxifen in tamoxifen-sensitive and tamoxifen-resistant human breast cancer cells. Anticancer Drugs 20, 723–735.](http://paperpile.com/b/SKkscP/THCF)

[Yin, F., Wakino, S., Liu, Z., Kim, S., Hsueh, W. A., Collins, A. R., et al. (2001). Troglitazone inhibits growth of MCF-7 breast carcinoma cells by targeting G1 cell cycle regulators. Biochem. Biophys. Res. Commun. 286, 916–922.](http://paperpile.com/b/SKkscP/1k2s)

[Zheng, H.-X., Wu, L.-N., Xiao, H., Du, Q., and Liang, J.-F. (2014). Inhibitory effects of dobutamine on human gastric adenocarcinoma. World J. Gastroenterol. 20, 17092–17099.](http://paperpile.com/b/SKkscP/2dkj)
